# Plant Architectural Structure and Leaf Trait Responses to Environmental Change: A Meta-Analysis

**DOI:** 10.3390/plants14111717

**Published:** 2025-06-04

**Authors:** Runze Li, Xiping Cheng, Pengyue Dai, Mengting Zhang, Minxuan Li, Jing Chen, Wajee ul Hassan, Yanfang Wang

**Affiliations:** 1 College of Soil and Water Conservation, Southwest Forestry University, Kunming 650224, China; lirunze227625@163.com (R.L.); xipingcheng@swfu.edu.cn (X.C.); zhangmengting0917@163.com (M.Z.); liminxuan0109@163.com (M.L.); 2 College of Gardening and Horticulture, Southwest Forestry University, Kunming 650224, China; chenjingswfu@gmail.com; 3 College of Forestry, Southwest Forestry University, Kunming 650224, China; wajeehassan2126@gmail.com

**Keywords:** environmental changes, plant architectural structure, leaf traits, meta-analysis, environmental factors

## Abstract

The relationship between plants and their environment has always been a core issue in ecological research. This study about how plant architecture and leaf traits respond to environmental changes helps to more deeply understand the adaptive mechanisms of plants in diverse environments. Although there have been related studies, a systematic analysis on a China-wide scale is still lacking. To address this gap, we conducted a meta-analysis of 115 studies across China examining plant architectural and leaf trait responses to environmental changes. The dataset includes 849 observations across 11 ecological variables, such as the mean annual precipitation, mean annual temperature, soil type, and elevation, and evaluates their effects on seven key plant traits. The results indicated that variations in the plant height, diameter at breast height (DBH), and root-to-shoot ratio are primarily influenced by the soil type and mean annual precipitation. In contrast, the soil type and mean annual sunshine duration mainly affected the specific leaf area (SLA), leaf area, leaf thickness, and leaf dry matter content. Moreover, while the magnitude of trait responses varies across precipitation, temperature, elevation, and soil property gradients, the impacts of environmental change are particularly pronounced under more extreme conditions. This study provides robust scientific evidence for understanding the effects of environmental change on plant growth across China and offers valuable insights into ecological conservation and the sustainable use of plant resources.

## 1. Introduction

The Intergovernmental Panel on Climate Change (IPCC) report predicted that by the end of the 21st century, global temperatures will rise by about 4 °C above pre-industrial levels, accompanied by significant shifts in global precipitation patterns. This climate change, characterized by a gradual increase in temperature and an increased frequency of extreme drought events, will affect the physiological functions of plants and may alter growth strategies and plant structures [1,2]. The interaction between plants and their environments has been a central topic in plant ecology research [3,4,5]. Investigating how plants adjust their architectural and functional traits in China’s highly diverse ecological environments in response to environmental change holds significant theoretical and practical value. Plant functional traits shaped by genetic factors and environmental conditions comprehensively reflect plant individuality and community structures, thereby revealing how plants adapt to environmental changes. The architectural structure of trees largely dictates their ability to compete and acquire resources, serving as a true reflection of their physiological processes and strategies for environmental adaptation [6]. Leaves are the plant organs most exposed to the atmospheric environment. As a crucial component of plant functional traits, leaf traits are strongly associated with plant biomass, resource acquisition, and utilization efficiency [7].

In this study, “environmental change” refers to the shift in growing conditions experienced by the same plant species across two distinct environments within China, where both conditions support healthy growth. Such environmental changes can manifest across multiple dimensions, including spatial heterogeneity and variation in resource availability. Specifically, spatial heterogeneity refers to the variability of environmental conditions across geographic space, such as differences in topography, soil properties, and plant community structures across regions, for instance, the pronounced environmental contrasts observed along elevation gradients. Resource heterogeneity manifests as uneven distributions of key ecological factors such as nutrients, water, and light. For example, soil nutrients often exhibit patchy spatial patterns, alternating wet and dry areas create moisture gradients, and variations in canopy density lead to significant differences in light availability within forest interiors.

The Spatial Variance Partitioning (SVP) hypothesis provides a theoretical framework for understanding the relative importance of intraspecific trait variation (ITV) and interspecific trait variation (BTV) across spatial scales. In this study, “large-scale” refers to plots located in regions more than 400 km apart, encompassing broader environmental gradients and greater geographic heterogeneity. In contrast, “small-scale” denotes plots within the same forest, grassland, or mountain, characterized by stronger spatial proximity and environmental similarity. From an ecological perspective, BTV tends to dominate at larger spatial scales due to significant species turnover across geographic locations, whereas ITV becomes more prominent at smaller scales where the species composition remains relatively stable. Therefore, we regard the SVP hypothesis as a conceptual framework to define our study’s categorization of habitat scales. In our analysis, ITV and BTV exhibited significant effects across scales, suggesting that transitions or overlaps between scales are common in real ecosystems and that both types of trait variation may coexist. Our definitions of “large” and “small” scales are grounded in the SVP framework, providing a theoretical basis for scale categorization in this study [8].

This study focuses on plant architecture and leaf functional traits. Plant height, the key component of phenotypic characteristics, influences biomass accumulation and vertical resource competition, while the stem diameter at breast height (DBH) is closely associated with growth patterns and biomass allocation. The leaf functional traits, such as specific leaf area (SLA), reflect the leaf resource investment strategy, the leaf area indicates the photosynthetic efficiency and transpiration characteristics, the leaf thickness embodies stress resistance and water retention capacity, and the leaf dry matter content reveals resource-use strategies and physiological–ecological characteristics. The root-to-shoot ratio comprehensively reflects the plant’s adaptive capacity for environmental changes [9,10,11,12,13,14,15].

Climatic factors serve as fundamental constraints for plant survival, with variables such as temperature, precipitation, and light jointly shaping the distribution of plant traits [16,17]. As topographic factors vary, such as the elevation, climate variables, including temperature, atmospheric pressure, precipitation patterns, and radiation intensity, undergo systematic changes, thereby regulating plant metabolic rates and life-history characteristics. In regions of China with pronounced north–south precipitation patterns, woody plants exhibit distinct canopy architectures and leaf physiological traits. In areas with pronounced elevation variations, such as the Hengduan Mountains in Yunnan Province, plant traits show systematic shifts along altitudinal gradients. For instance, the leaf thickness tends to increase with elevation. This pattern is consistent with findings from a study of 118 plant species on New Zealand’s South Island [18]. Meanwhile, soil texture and physio-chemical properties influence plant resource availability and physiological adaptability. For example, plants growing in southern China’s nutrient-poor acidic red soils commonly exhibit smaller leaf areas. This phenomenon reflects a pattern of ecological convergence observed in 88 species of the genus *Leucadendron* from the coastal regions of southern Africa [19].

Scholars have conducted extensive research on the effects of environmental changes on plant morphology and functional traits. These studies mainly focus on how environmental factors, such as temperature, moisture, and soil properties, influence plant growth and distribution [20,21,22]. Temperature is a significant factor affecting plant growth [16]. A study conducted in China revealed that leaf thickness in C4 plants increases with rising temperatures, whereas C3 plants exhibit no consistent pattern in the leaf thickness response. Precipitation also exerts significant effects on plant traits [23]. In the arid regions of northern China, although precipitation is a limiting factor, its impact on plant growth is inconsistent. For instance, increased annual rainfall does not consistently promote height growth in *Caragana shrubs*, and the effect varies across years and depends on topographic conditions [24]. Soil is a crucial component of terrestrial ecosystems, providing the essential environment for plant growth. A study on *Picea schrenkiana* in Xinjiang, China, found that soil moisture content and soil nitrogen availability are the primary drivers of variation in its leaf functional traits along an elevation gradient. In contrast, studies on forest plants in the karst ecosystems of southwestern China have found a significant negative correlation between soil moisture content and leaf dry matter content [25,26].

In summary, although studies on the relationships between plant functional traits and environmental factors in China have increased in recent years and yielded numerous regional-level findings, due to differences in spatial and temporal distribution, random biases, plant types, and research priorities, it does not remain easy to establish a universally applicable theoretical framework across diverse study environments. Currently, most studies conducted in China focus on specific regions or single species, with relatively few comparative analyses across large spatial scales and multiple plant types, resulting in a lack of systematic cross-regional comparisons. Consequently, future research must adopt a broader framework incorporating multiple regions, large spatial scales, diverse species, and interactive environmental factors to better understand how ecological change shapes plant adaptive mechanisms and biodiversity patterns. Building on China’s diverse environmental contexts and abundant field research data, this study employed a meta-analytic approach to systematically synthesize literature from across China, focusing on the responses of seven representative plant traits to eleven key environmental variables across various climate zones and ecologically representative regions. We aim to provide empirical evidence from a Chinese perspective to elucidate the evolutionary logic of plant ecological strategies under climate change. We aim to provide empirical evidence from a Chinese perspective to uncover the evolutionary logic of plant ecological strategies under climate change and to offer scientific support for ecosystem conservation, biodiversity maintenance, and environmental engineering practices in China.

## 2. Materials and Methods

### 2.1. Data Sources

**Figure 1:**
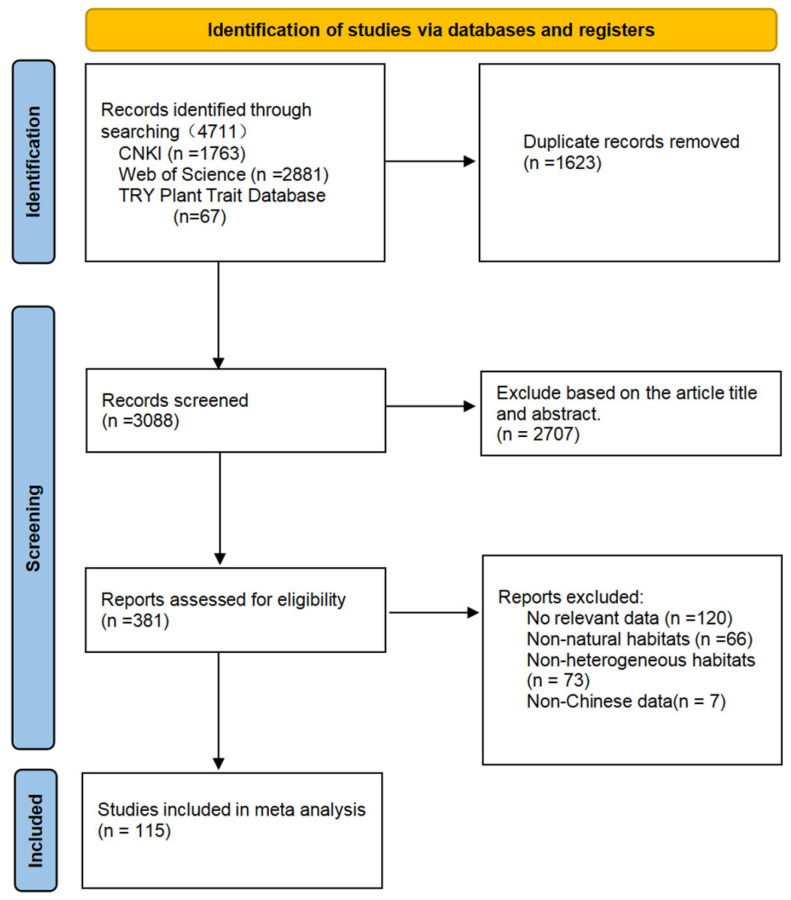
Workflow of selected sources of information.

To investigate how environmental changes affect the plant structure and functional traits, we used the workflow of detecting and selecting sources of information via the preferred reporting items for systematic reviews and meta-analyses (PRISMA) (Figure 1). This study utilized academic databases, including CNKI (www.cnki.net) (accessed on 1 May 2024), Web of Science (https://www.webofscience.com/wos/) (accessed on 1 May 2024), TRY Plant Trait Database (https://www.try-db.org) (accessed on 1 May 2024) [27], and other Chinese and English databases, for literature retrieval focused on studies published up to 1 May 2024. The references and plant species involved in this study are provided in Supplementary Materials S2 and S3. The titles, abstracts, and keywords included search terms such as “different environments” OR “heterogeneous environments,” OR “different habitats,” OR “heterogeneous habitats,” OR “environment change,” AND “plant functional traits” OR “morphological characteristics” OR “Tree structure” OR “variation.” After removing duplicates, screening titles and abstracts, and applying standard criteria, 115 studies meeting the inclusion criteria were selected for the meta-analysis. (1) The plant species discussed in the article must grow in at least two environments. (2) The literature must reference one or more of the following plant traits: tree height (plant height), diameter at breast height, leaf area, specific leaf area, leaf thickness, root-to-shoot ratio, and leaf dry matter content; (3) the literature must provide adequate statistical information including the mean and standard deviation (or standard error) for at least one of the indicators from the plant height, diameter at breast height, leaf area, specific leaf area, leaf thickness, root-to-shoot ratio, and leaf dry matter content under environmental changes to allow for the calculation of effect sizes; (4) the subjects of the study must be plant communities in natural ecosystems with artificially controlled experimental studies excluded from the analysis.

### 2.2. Data Categorization

In research examining the response of the plant architecture and leaf functional traits to environmental changes, we divided the database into 11 groups to compare how environmental changes across experimental regions, plant species, and ecosystems affect plants due to significant differences in environmental factors. We classified and organized the data using multiple approaches based on their distribution range, relevant literature, and the practical context of this study. To investigate the impact of environmental factors at the study sites on plants, we extracted data on climate, terrain, and soil characteristics from the experimental plots, including the soil type, soil organic matter, soil moisture, climate type, elevation, relative humidity, annual average temperature, annual precipitation, and annual sunlight duration. If the original literature did not report the elevation of the study site, we extracted the corresponding elevation data using the geographic coordinates provided based on the digital elevation model (DEM) from the WorldClim2 dataset. Other climatic and soil variables were obtained directly from the original studies; if such information was missing, we supplemented the data using ecological studies conducted in the same region and period to ensure temporal and spatial consistency. For plant functional group (PFG) classification, we adopted the categories provided in the original literature when they were available; otherwise, we classified species according to China’s National Specimen Information Infrastructure (NSII). Furthermore, we standardized the variables reported in different units in the literature using conversion formulas.

In summary, we compiled a dataset containing 849 paired observations from 115 articles examining the effects of environmental change on plant growth. This dataset includes 156 pairs for the specific leaf area, 60 for the stem diameter at breast height (DBH), 217 for the plant height, 108 for the leaf thickness, 160 for the leaf area, 97 for the leaf dry matter content, and 51 for the root-to-shoot ratio. In selecting classification criteria, we followed the methods used in other ecological meta-analyses, categorizing data by gradients like annual precipitation, temperature, elevation, and soil moisture content [28,29]. This classification method accurately reflects the plant architecture and responses across various environmental gradients. Climate classification was based on the Köppen system, a widely recognized method that effectively demonstrates the influence of different climate types on plant growth. As this study was based on nationwide surveys across China, soil types were classified according to the Chinese Soil Taxonomy to ensure scientific accuracy and practical applicability. Soil organic matter content was divided into four levels based on the classification standards from China’s Second National Soil Survey [30]. Annual sunshine duration was categorized into three levels according to the mean annual sunshine hours: low (0–1500 h), medium (1500–2500 h), and high (>2500 h). Relative humidity was classified into arid (0–60%) and humid (60–100%) based on regional environmental moisture conditions. This classification strategy balances ecological relevance with statistical feasibility, ensuring that subgroup differences are interpretable and sample sizes are sufficient. (Table 1).

### 2.3. Data Retrieval

**Figure 2:**
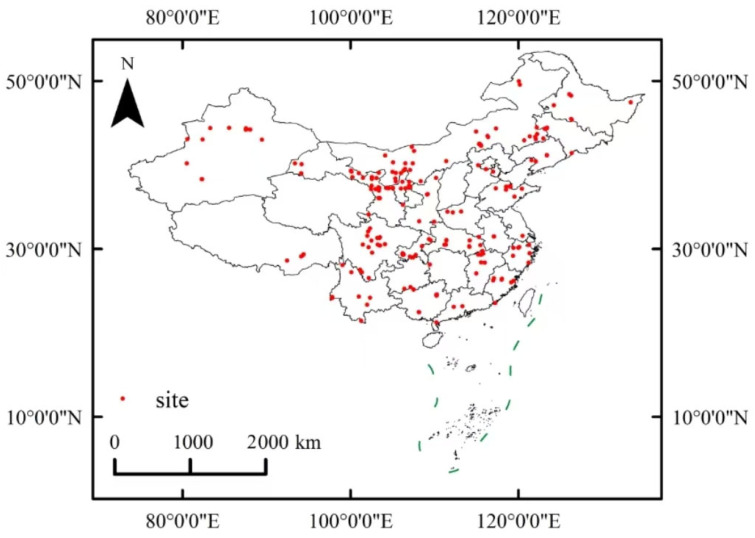
Distribution of research area points from data sources considered in the present study.

A meta-analysis requires the sample size, mean, and standard deviation (Standard Deviation, *SD*) under control and treatment conditions. This study used WebPlotDigitizer (Version 4.5) software to extract this data from charts. This software’s scientific reliability and accuracy have been validated [31]. Table data were directly extracted and integrated in the form of the mean (*X*), standard deviation (*SD*), and sample size (*n*) for control and experimental groups. We illustrate the distribution and collection of study points in Figure 2.

If an indicator is presented as a standard error (*SE*), the conversion is carried out using the following formula.

(1) $$SD=SE\sqrt{n}$$

In the equation, *SD* stands for the standard deviation, *SE* stands for the standard error, and n stands for the sample size. Some articles do not specify the number of leaves when testing leaf functional traits. In these cases, this study uses the mean value.

Effect sizes provide a good measure of the treatment effects on indicators and quantify the relationships between variables. In plant ecology, the response ratio (*RR*) has been extensively used in meta-analyses [32]. Therefore, this study uses the natural logarithm of the *RR*, as proposed by Hedges et al. in 1999, as the effect size [33]. The formula is as follows:

(2) $$RR=InR=In\frac{Xt}{Xc}=InXt-InXc$$

In the formula, *RR*, *R*, *X_t_*, and X_c_ stand for the effect size, response ratio, mean value of the treatment group, and mean value of the control group, respectively.

(3) $$v=\frac{{\mathrm{SD}}_{t}^{2}}{X_{t}^{2}n_{t}}+\frac{SD_{c}^{2}}{X_{c}^{2}n_{c}}$$

The effect sizes of all results were weighted using the non-parametric weight factor w, which is the inverse of the variance v:

(4) $$w=\frac{1}{v}$$

*I*n*RR*_++_ and *SD_InRR++_* are the weighted mean effect size and variance after non-parametric weighting, with the calculation formulas as follows:

(5) $$InRR_{++}=\frac{\sum In\;R_{i}\;{\times \;w}_{i}\;}{\sum w_{i}}$$

(6) $${\mathrm{SD}}_{{\mathrm{InRR}}_{++}}=\sqrt{\frac{1}{\sum W_{i}}}$$

This study uses a random effects model to calculate the 95% confidence interval of effect sizes, extracting *R*_++_ and the Bootstrap CI for each indicator under environmental changes. If the confidence interval includes 0, it suggests that environmental changes do not significantly affect the plant architecture and leaf functional traits. If the confidence interval excludes 0, it indicates a significant impact of environmental changes on the plant architecture and leaf functional traits. (*p* < 0.05).

### 2.4. Statistical Evaluation

This study utilized OpenMEE software (http://www.cebm.brown.edu/openmee/) (accessed on 1 June 2024) to conduct the meta-analysis of the different types of data. To evaluate the impact of various environmental factors on the plant architectural structure and functional traits, the “rfPermute” package was implemented for random forest analysis. Using this approach, the percentage increase in mean square error (%IncMSE) was utilized as the metric to rank the importance of each influencing factor. Data statistical analysis for this study was carried out using R 4.4.0 software.

## 3. Results

### 3.1. Subgroup Analysis of Plant Architectural Traits Responding to Environmental Change

**Figure 3:**
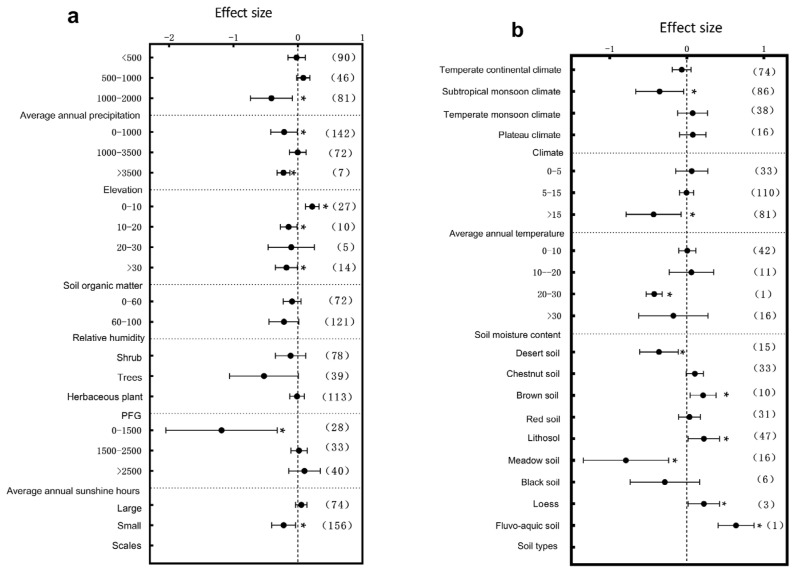
Subgroup analysis of plant height in response to environmental changes. If the 95% confidence interval does not overlap with 0, the effect is considered significant; the values in parentheses represent the sample size, and (*) indicates significance at the 0.05 level. (**a**) Subgroup analyses of average annual precipitation, elevation, soil organic matter, relative humidity, scale, PFG, and annual sunshine hours; (**b**) subgroup analyses of climate types, average annual temperature, soil moisture content, and soil type.

**Figure 4:**
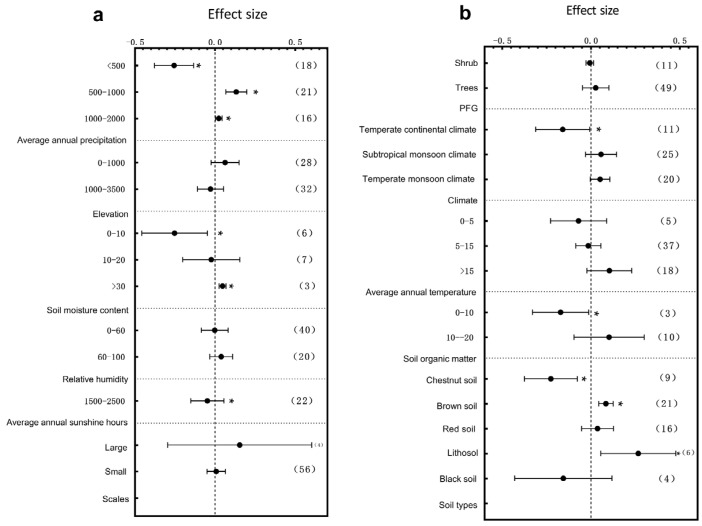
Subgroup analysis of DBH in response to environmental changes. If the 95% confidence interval does not overlap with 0, the effect is considered significant; the values in parentheses represent the sample size, and (*) indicates significance at the 0.05 level. (**a**) Subgroup analyses of average annual precipitation, elevation, soil moisture, relative humidity, scale, and annual sunshine hours; (**b**) subgroup analyses of PFG, climate types, average annual temperature, soil organic matter content, and soil type.

**Figure 5:**
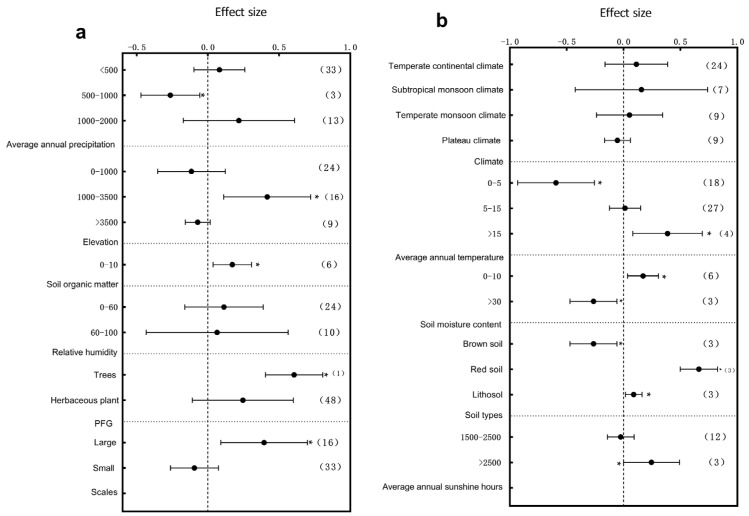
Subgroup analysis of root-to-shoot ratio in response to environmental changes. If the 95% confidence interval does not overlap with 0, the effect is considered significant; the values in parentheses represent the sample size, and (*) indicates significance at the 0.05 level. (**a**) Subgroup analyses of average annual precipitation, elevation, soil organic matter content, relative humidity, scale, annual Sunshine Hours, and PFG; (**b**) subgroup analyses of climate types, average annual temperature, soil moisture, and soil type.

The influence of climatic factors on plant growth is very crucial. In terms of annual precipitation, different precipitation intervals exerted significant effects on the plant height, diameter at breast height (DBH), and root-to-shoot ratio. Specifically, the plant height responded most significantly to environmental variation within the 1000–2000 mm annual precipitation range (Figure 3a, *p* < 0.05); DBH showed significant variation across the 0–2000 mm range with particularly pronounced changes in the 0–500 mm interval (Figure 4a, *p* < 0.05), and the root-to-shoot ratio responded significantly within the 500–1000 mm range (Figure 5a, *p* < 0.05). These results indicate that precipitation, a crucial water resource, significantly regulates variations in plant growth traits within specific ranges. The annual sunshine duration and mean annual temperature also considerably influenced the responses of plant traits to environmental changes. The plant height was significantly affected in regions with an annual sunshine duration of 0–1500 h (Figure 3a, *p* < 0.05); regarding the mean annual temperature, plant height exhibited significant responses only in areas with temperatures >15 °C (Figure 3b, *p* < 0.05), while the root-to-shoot ratio showed significant variation under both 0–5 °C and >15 °C conditions (Figure 5b, *p* < 0.05). Climatic types, as integrative climatic factors, also pose differentiated regulatory effects. Environmental changes in subtropical monsoon climate regions significantly influenced plant height (Figure 3b, *p* < 0.05), whereas temperate continental climates primarily affected DBH (Figure 4b, *p* < 0.05), and no significant trait variations were observed under other climate types.

Soil physio-chemical properties also exert substantial effects on the plant architecture. Further analysis revealed that soil organic matter content variations significantly regulated trait expressions. Plant height was significantly influenced by soil organic matter contents of 0–10 g/kg^−1^, 10–20 g/kg^−1^, and >30 g/kg^−1^, with the most pronounced changes observed in the 0–10 g/kg^−1^ range (Figure 3a, *p* < 0.05). DBH was significantly affected only within the 0–10 g/kg^−1^ range (Figure 4b, *p* < 0.05), while the root-to-shoot ratio also showed significant responses in areas with low organic matter (0–10 g/kg^−1^) (Figure 5a, *p* < 0.05). Besides resource supply factors, the environmental scale also significantly affected plant trait variations. Results showed that small-scale environmental changes significantly influenced the plant height (Figure 3a, *p* < 0.05), whereas the root-to-shoot ratio was more sensitive to environmental variations in large-scale habitats (Figure 5a, *p* < 0.05). Regarding the soil moisture content, plant height was significantly affected within the 20–30% range (Figure 3b, *p* < 0.05), DBH exhibited significant variation in both the 0–10% and >30% ranges (Figure 4a, *p* < 0.05), and the root-to-shoot ratio was significantly regulated under extremely low (0–10%) and extremely high (>30%) soil moisture conditions (Figure 5b, *p* < 0.05). The soil type also largely determined the patterns of plant trait responses to environmental changes. The plant height exhibited significant variations in brown soils, loess, lithosol, fluvo-aquic soils, desert soils, and meadow soils (Figure 3b, *p* < 0.05); DBH varied significantly under all soil types except red soils and black soils (Figure 4b, *p* < 0.05), while the root-to-shoot ratio was mainly influenced by brown soils, red soils, and lithosol (Figure 5b, *p* < 0.05). Finally, an elevation gradient analysis revealed that, except for plant height within the 1000–3500 m range where no significant differences were observed, all other elevation intervals exhibited significant trait responses, with plant height showing the most pronounced variation at >3500 m (Figure 3a, *p* < 0.05). The root-to-shoot ratio was also significantly affected in the mid-to high-elevation range (1000–3500 m) (Figure 5a, *p* < 0.05), highlighting the spatial heterogeneity effects of topographical undulations on plant growth responses. Overall, the sensitivities of the plant height, DBH, and root-to-shoot ratio to environmental changes are jointly driven by multiple natural factors. Different traits exhibited heterogeneous response patterns under the influence of diverse ecological factors, reflecting the adaptive characteristics of plants to complex environmental gradients.

### 3.2. Subgroup Analysis of Plant Leaf Traits Responding to Environmental Change

**Figure 6:**
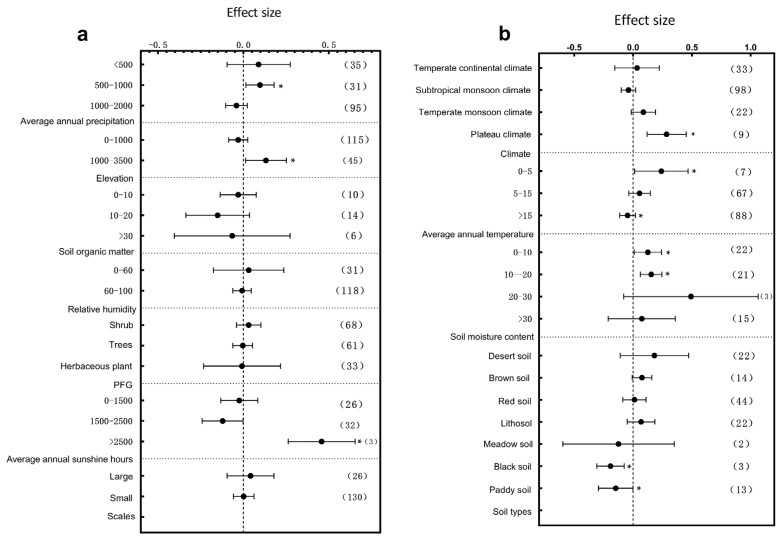
Subgroup analysis of specific leaf area in response to environmental changes. If the 95% confidence interval does not overlap with 0, the effect is considered significant; the values in parentheses represent the sample size, and (*) indicates significance at the 0.05 level. (**a**) Subgroup analyses of average annual precipitation, elevation, soil organic matter, relative humidity, scale, PFG, and annual sunshine hours; (**b**) subgroup analyses of climate types, average annual temperature, soil moisture content, and soil type.

**Figure 7:**
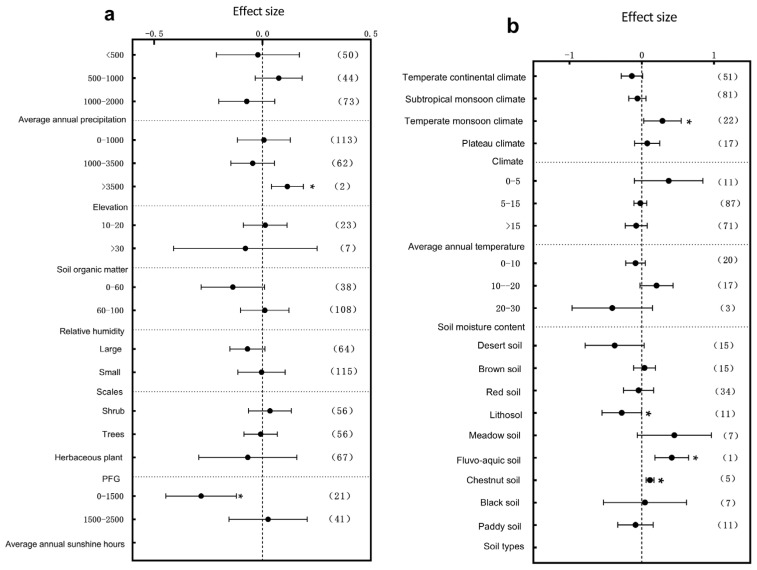
Subgroup analysis of leaf area in response to environmental changes. If the 95% confidence interval does not overlap with 0, the effect is considered significant; the values in parentheses represent the sample size, and (*) indicates significance at the 0.05 level. (**a**) Subgroup analyses of average annual precipitation, elevation, soil organic matter, relative humidity, scale, PFG, and annual sunshine hours; (**b**) subgroup analyses of climate types, average annual temperature, soil moisture content, and soil type.

**Figure 8:**
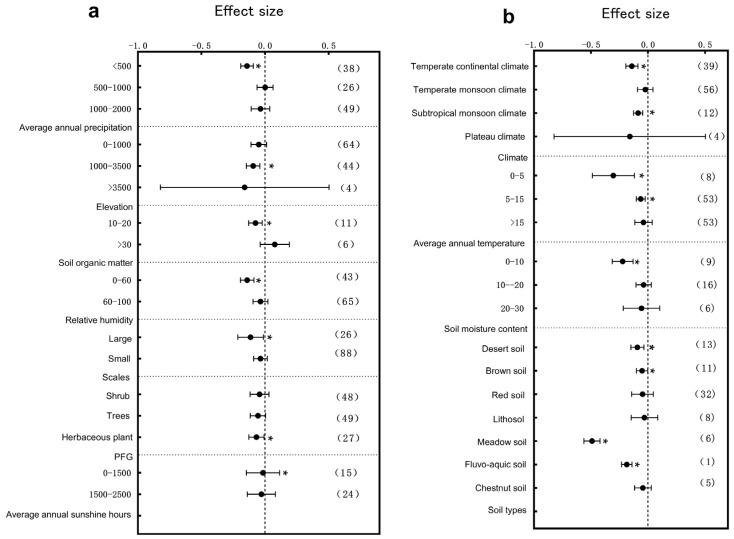
Subgroup analysis of leaf thickness in response to environmental changes. If the 95% confidence interval does not overlap with 0, the effect is considered significant; the values in parentheses represent the sample size, and (*) indicates significance at the 0.05 level. (**a**) Subgroup analyses of average annual precipitation, elevation, soil organic matter, relative humidity, scale, PFG, and annual sunshine hours; (**b**) subgroup analyses of climate types, average annual temperature, soil moisture content, and soil type.

**Figure 9:**
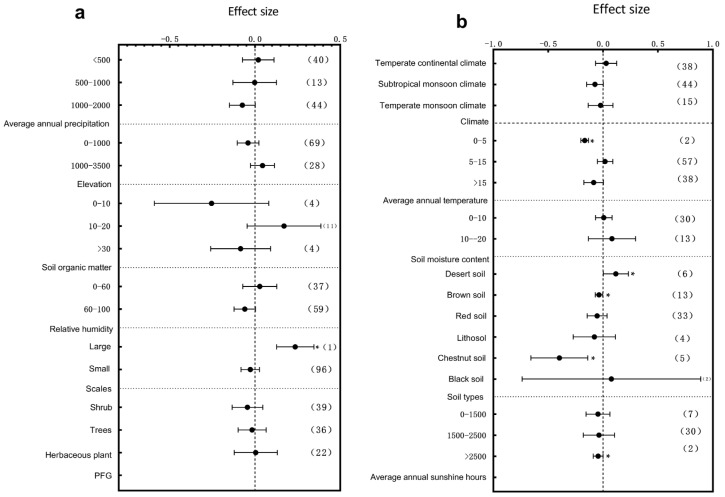
Subgroup analysis of leaf dry matter content in response to environmental changes. If the 95% confidence interval does not overlap with 0, the effect is considered significant; the values in parentheses represent the sample size, and (*) indicates significance at the 0.05 level. (**a**) Subgroup analyses of average annual precipitation, elevation, soil organic matter, relative humidity, scale, and PFG; (**b**) subgroup analyses of climate types, average annual temperature, soil moisture content, soil type, and annual sunshine hours.

The specific leaf area (SLA), leaf area, leaf thickness, and leaf dry matter content exhibited significant and differentiated response patterns to environmental changes under the influence of multiple environmental factors. First, from the water resources perspective, different mean annual precipitation ranges played a key regulatory role in trait responses. SLA was significantly influenced by environmental changes within the 500–1000 mm precipitation range (Figure 6a, *p* < 0.05), while leaf thickness was most sensitive under arid conditions (<500 mm) (Figure 8a, *p* < 0.05). Further analysis along the temperature gradient revealed that trait responses varied with thermal conditions. SLA was significantly affected in regions where the mean annual temperature ranged between 0–5 °C and >15 °C (Figure 6b, *p* < 0.05), with the magnitude of the response decreasing with the increase in temperature. Leaf thickness showed significant variations in 0–5 °C and 5–15 °C intervals (Figure 8b, *p* < 0.05), with more pronounced differences observed within the 0–5 °C range. The leaf dry matter content mainly exhibited significant responses to environmental changes in cold regions (0–5 °C) (Figure 9b, *p* < 0.05). Light conditions also played an essential role in trait regulation. Both the SLA and leaf dry matter content were significantly influenced by environmental variation in areas with an annual sunshine duration of >2500 h (Figure 6a and Figure 9b, *p* < 0.05), whereas the leaf area showed significant changes under low sunshine conditions ranging as (0–1500 h) (Figure 7a, *p* < 0.05). Moreover, climate variation differences partly shaped plant trait response patterns. The leaf area was significantly influenced by environmental variation under temperate monsoon climates (Figure 7b, *p* < 0.05), while leaf thickness showed significant responses under both temperate continental and subtropical monsoon climates (Figure 8b, *p* < 0.05). No prominent trait responses were observed under other climate types. Meanwhile, the plant functional group (PFG) also played an essential role in regulating leaf thickness variations, with herbaceous plants exhibiting extreme sensitivity to environmental changes (Figure 8a, *p* < 0.05).

Regarding the soil physio-chemical properties, soil organic matter content, soil type, and soil moisture collectively influenced plant trait responses to environmental variation. The leaf thickness was significantly affected in regions with soil organic matter content between 10 and 20 g/kg^−1^ (Figure 8a, *p* < 0.05). Under different soil types, SLA showed significant responses to environmental changes, mainly in paddy soils and black soils (Figure 6b, *p* < 0.05); the leaf area varied significantly in fluvo-aquic soils, chestnut soils, and Lithosol (Figure 7b, *p* < 0.05); the leaf thickness exhibited significant variations in desert soils, meadow soils, fluvo-aquic soil, and brown soils (Figure 8b, *p* < 0.05). The leaf dry matter content responded significantly to environmental changes in desert, brown, and chestnut soils (Figure 9b, *p* < 0.05). Consistent with precipitation variations, soil moisture also played a crucial role. Soil moisture levels significantly influenced SLA within the 0–10% and 10–20% ranges (Figure 6b, *p* < 0.05), while leaf thickness responded most strongly under extremely low-soil-moisture conditions in the range of (0–10%) (Figure 8b, *p* < 0.05). Changes in the environmental scale further regulated plant trait responses. Larger environmental scale variations significantly affected the leaf thickness and dry matter content (Figure 8a and Figure 9a *p* < 0.05), indicating that macro-scale heterogeneity is vital in shaping plant adaptive strategies. Finally, an analysis of topographical factors revealed that elevation gradients exerted differential regulatory effects on trait variations. SLA was significantly affected within the 1000–3500 m elevation range (Figure 6a, *p* < 0.05), whereas the leaf area showed the most pronounced variation at elevations >3500 m (Figure 7a, *p* < 0.05). The leaf thickness also exhibited significant responses to environmental changes within the mid-to-high elevation range of 1000–3500 m (Figure 8a, *p* < 0.05). In summary, the combined effects of precipitation, elevation, sunshine duration, temperature, soil physio-chemical properties, climate types, and environmental scale jointly drove the heterogeneous responses of the SLA, leaf area, leaf thickness, and leaf dry matter content across different ecological environments, reflecting the fine-scale adaptive mechanisms of plants under complex environmental changes.

### 3.3. Primary Factors Influencing the Plant Structure and Leaf Traits

**Figure 10:**
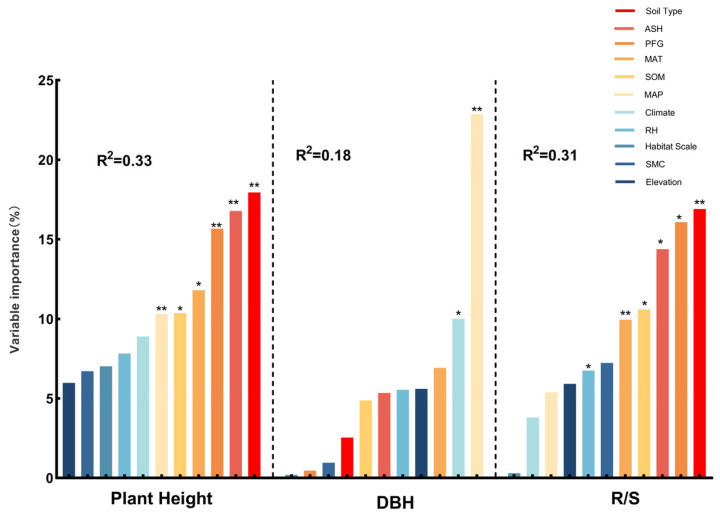
The main factors affecting the effect values of environmental changes on plant height, diameter at breast height, and root-to-shoot ratio. (*) indicates *p* < 0.05, (**) indicates *p*< 0.01. When *p* < 0.05, it indicates a significant effect of the environmental factor on plant traits, while *p* < 0.01 indicates an extremely significant effect. The value of R^2^ ranges from 0 to 1. As R^2^ increases, the explanatory power of the model also increases. Plant height, *n* = 217; diameter breast height (DBH), *n* = 60; root-to-shoot ratio (R/S), *n* = 49. ASH (annual sunshine hours), MAT (mean annual temperature), SOM (soil organic matter), MAP (mean annual precipitation), RH (relative humidity), SMC (soil moisture content), PFG (plant functional group).

The effect sizes of plant architectural traits under varying environmental conditions were primarily influenced by the soil type and mean annual precipitation. The effect size of tree height is affected mainly by the soil type (17.94%) and annual sunshine duration (16.78%) (Figure 10), with other major influencing factors ranked by explanatory power, such as the plant functional groups (PFGs), annual mean temperature, soil organic matter, and annual precipitation. Conversely, the effect size of diameter at breast height (DBH) is mainly determined by the annual precipitation (22.87%) and climate type (10%) (Figure 10), with other key factors ranked by explanatory power, such as the annual mean temperature, elevation, and relative humidity. The effect size for the root-to-shoot ratio was mainly determined by the soil type (16.91%) and plant functional groups (PFGs) (16.08%) (Figure 10), with additional contributions from the mean annual sunshine duration, soil organic matter content, and mean annual temperature.

**Figure 11:**
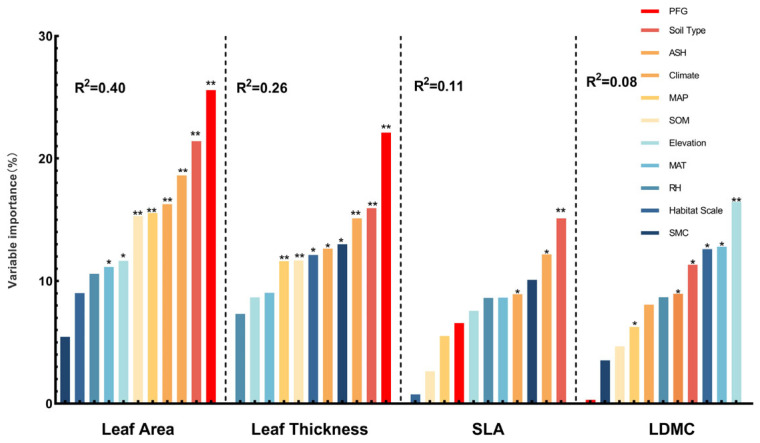
The main factors affecting the effect values of environmental changes on leaf area, leaf thickness, specific leaf area, and leaf dry matter content. (*) indicates *p* < 0.05, (**) indicates *p* < 0.01. When *p* < 0.05, it indicates a significant effect of the environmental factor on plant traits, while *p* < 0.01 indicates an extremely significant effect. The value of R^2^ ranges from 0 to 1; as R^2^ increases, the explanatory power of the model also increases. Leaf area, *n* = 160; leaf thickness, *n* = 108; specific leaf area (SLA), *n* = 156; leaf dry matter content (LDMC), *n* = 97. ASH (annual sunshine hours), MAT (mean annual temperature), SOM (soil organic matter), MAP (mean annual precipitation), RH (relative humidity), SMC (soil moisture content), PFG (plant functional group).

The effect sizes of plant leaf traits under varying environmental conditions were primarily influenced by the soil type and mean annual sunshine duration. The effect size of the leaf area is affected mainly by the plant functional groups (PFGs) (25.59%) and soil type (21.42%) (Figure 11), with other major influencing factors ranked by explanatory power, such as the annual sunshine duration, climate, and annual precipitation. The effect size of leaf thickness is mainly influenced by the plant functional groups (PFGs) (22.11%) and soil type (15.94%) (Figure 11), with other key factors ranked by explanatory power, such as the annual sunshine duration, soil moisture content, and climate. The effect size of the specific leaf area (SLA) is primarily determined by the soil type (15.12%) and annual sunshine duration (12.17%) (Figure 11), with additional influencing factors, in descending order of explanatory power, such as the soil moisture content, climate, and annual mean temperature. The effect size for the leaf dry matter content was primarily driven by the elevation (16.46%) and mean annual temperature (12.80%) (Figure 11), with additional influences from the habitat scale and soil type.

## 4. Discussion

### 4.1. The Response of Plant Architectural Structure to Environmental Changes

The architectural structure of plants is shaped not only by genetic traits but also by external environmental factors [34]. A random forest analysis indicated that the soil type and mean annual precipitation are the primary ecological drivers influencing plant architecture (Figure 10). Soil properties regulate plant growth by affecting water retention, mechanical support, and nutrient availability [35]. Studies conducted in northeastern China have shown that precipitation gradients are a major driver of variation in the stem diameter; plants growing in high-precipitation areas exhibit larger stem diameters, reflecting the promotion of growth rates and carbon accumulation by water availability [36]. As the changes are anticipated in precipitation patterns and soil properties, dynamic adjustments of plant architecture may have profound implications for ecosystem stability.

Water is a vital survival resource and is key to regulating trait variation. Our study revealed that the plant height responded most sensitively to environmental changes in regions with moderate precipitation (1000–2000 mm) (Figure 3a), whereas the DBH and root-to-shoot ratio exhibited the most significant variation in arid areas (0–500 mm) (Figure 4a and Figure 5a), This reflects divergent resource-allocation strategies evolved under different moisture conditions [37]. Furthermore, under extreme soil moisture conditions (0–10% and >30%), the DBH and root-to-shoot ratio showed significant variation (Figure 4a and Figure 5b). This supports the edge adaptation strategy, which states that extreme stress accelerates functional trait diversification [38]. An analysis of satellite-derived soil moisture data across various soil types in China has revealed that areas with extreme soil moisture levels are ecologically sensitive zones. Plants must adapt by modifying their root architecture and growth rates to enhance resource acquisition and survival capacity [39]. Studies in southwestern China’s karst region have shown that topography-induced soil moisture gradients significantly influence the resource-allocation strategies of Rhus chinensis. This phenomenon suggests that plants adopt “resource-conserving” strategies in water-limited environments, such as reducing transpiration to cope with drought stress, highlighting divergent evolutionary adaptations in resource allocation under moisture heterogeneity. These findings provide empirical support for understanding plant adaptive mechanisms along moisture gradients at the national scale in China [40]. As global drought intensifies, drought-tolerant species characterized by high root-to-shoot ratios may increasingly dominate communities, reshaping the ecosystem structure and function.

Energy inputs such as heat and light also significantly shape plant traits. We found that the root-to-shoot ratio varied significantly under extreme thermal conditions (0–5 °C and >15 °C) (Figure 5b), with high temperatures promoting greater root investment to alleviate water deficits and cold conditions favoring underground biomass storage [41,42]. A related study found that under rising temperature conditions on the Qinghai–Tibet Plateau, two alpine meadow plants adopted adaptive strategies by increasing their belowground biomass, reflecting a dual response to soil moisture stress and warming. Specifically, *Kobresia humilis* exhibited greater carbon allocation to roots and deeper root development, whereas *Potentilla fruticosa* responded more conservatively. These divergent resource-allocation strategies reflect the diverse adaptive mechanisms of plants to climate warming across different alpine ecosystems [43]. This evolutionary strategy reflects the long-term optimization of resource stability and survival probability. Along the light gradient, plant height responded significantly to environmental variation in low-light regions (<1500 h) (Figure 3a), highlighting the ability of plants to adjust their morphology and resource allocation according to the energy supply [44]. *Phoebe bournei* exhibits a low-plasticity yet physiologically regulated resource-allocation strategy in the varying light environments of subtropical humid forests in China. Rather than relying on dynamic adjustments in resource allocation among roots, stems, and leaves, the species adapts to environmental variation by regulating photosynthetic parameters and energy expenditure [45]. Future climate warming and radiation changes are expected to shift the balance between fast-growing and conservative species, altering ecosystem energy flow patterns.

Soil physio-chemical properties fundamentally determine plant resource acquisition [46,47]. Our results showed that in low-organic-matter soils (0–10 g/kg^−1^), the plant height, DBH, and root-to-shoot ratio varied significantly (Figure 3a, Figure 4b and Figure 5a). Plants tend to reduce above-ground biomass and enhance root development, which is considered a widely evolved resource-conservative strategy in nutrient-poor environments [48,49]. A meta-analysis conducted at the national scale in China revealed that under increased soil nutrient availability, plants in terrestrial ecosystems exhibit a pronounced “aboveground-prioritized” resource-allocation strategy, channeling more biomass to aboveground organs to enhance photosynthetic efficiency. In contrast, when soil nutrients are limited, plants allocate more resources to root development to improve water and nutrient acquisition [50]. Environmental changes have a pronounced impact on architectural structures under different soil types, particularly in brown soil and lithosol regions (Figure 3b, Figure 4b and Figure 5b). This supports the idea that soil heterogeneity drives local adaptation [51]. Elevation gradients profoundly affect plant trait divergence by regulating the temperature, radiation, moisture, and soil properties. Our study found that plant height varied significantly at elevations >3500 m, while the root-to-shoot ratio responded significantly at 1000–3500 m (Figure 3a and Figure 5a). This suggests that plants adjust above- and below-ground resource allocation to adapt to extreme conditions [52,53]. Plants exhibit distinct resource-allocation strategies in grassland ecosystems across different elevations in China at the individual level. Alpine plants on the Qinghai–Tibet Plateau at high elevations tend to allocate more resources to aboveground structures to cope with extreme conditions such as low temperatures, high radiation, and short growing seasons. In contrast, temperate grassland plants on the lower-elevation Inner Mongolia Plateau prioritize belowground expansion to enhance water and nutrient uptake. This divergence in aboveground–belowground biomass allocation reflects the long-term ecological adaptation of plants to elevation gradients and associated environmental factors [54]. Harsh alpine environments have promoted the evolutionary development of stable adaptive strategies through long-term natural selection. Due to strong spatial gradients and micro-habitat isolation, mountain systems serve as hotspots for plant diversity evolution [55], such as in the Hengduan Mountains of China and the Andes of South America. However, climate warming may disrupt these patterns, reshaping mountain ecosystem structures.

A 2021 global meta-analysis of alpine plants demonstrated that drought exerted a far more substantial inhibitory effect on plant growth than the effect of increased precipitation, with inter-specific variation in drought sensitivity. Consistently, our study found that the DBH and root-to-shoot ratio were significantly affected under drought conditions (Figure 4a and Figure 5a). Importantly, our study revealed spatial heterogeneity in trait responses across elevation gradients [56]. A 2022 global meta-analysis on forest-scale root-to-shoot ratios also showed sensitivity to water availability but focused primarily on warm and humid regions; our results complement these findings by providing evidence from cold alpine environments [57]. Overall, this study confirms the high sensitivity of root-to-shoot ratios to multiple environmental factors and highlights the distinct responses in cold regions.

### 4.2. The Response of Leaf Traits to Environmental Changes

Plant leaf functional traits are susceptible to climate change, reflecting their strong capacities for environmental adaptation [58,59]. Recent global studies have shown that at large spatial scales, the leaf trait variation is predominantly controlled by climatic factors, whereas at local scales, soil attributes exert a greater influence [60,61]. A random forest analysis further revealed that compared with plant architectural traits, leaf functional characteristics are significantly influenced by the soil type and annual sunshine duration (Figure 11). Soil properties mainly regulate the specific leaf area (SLA), leaf thickness, and leaf dry matter content (LDMC). This highlights their importance in determining plant resource allocation. In contrast, the sunshine duration significantly affects the leaf area and SLA, suggesting that plants adjust their leaf structure to optimize energy capture [62].

Water availability emerged as a key driver of leaf trait variation. This study found that SLA varied significantly in regions where the annual precipitation ranged between 500 and 1000 mm (Figure 6a), while extreme drought conditions (<500 mm) induced the most significant variation in leaf thickness (Figure 8a). Similarly, low soil water content (0–10%) triggered synchronous changes in SLA and leaf thickness (Figure 6b and Figure 8b). Drought selection pressures promote the evolution of low SLA and increased leaf thickness, leading to enhanced water retention and energy use efficiency [63]. In the desert ecosystems of the Hexi Corridor in China, natural precipitation gradients drive typical desert shrubs to adjust a suite of leaf functional traits, reflecting distinct adaptive strategies in resource allocation. These adaptations include a reduced specific leaf area, increased leaf thickness, and higher tissue density, contributing to the formation of structurally stable and metabolically efficient leaf systems. This conservative, drought-tolerant strategy represents an evolutionary adaptation to arid conditions and underscores the critical role of moisture gradients in shaping leaf functional traits across China [64]. Plants with such traits gain survival advantages under prolonged drought, reflecting directional trait evolution under water stress [65]. With future global aridification trends, the selection pressure toward more conservative functional strategies is expected to intensify.

Thermal conditions also profoundly shape plant metabolism and growth, thus driving the evolution of leaf traits. We found that in cold regions with a mean annual temperature of 0–5 °C, the SLA, leaf thickness, and LDMC showed significant responses to environmental variation (Figure 6b, Figure 8b and Figure 9b), with leaf thickness being the most sensitive trait. In contrast, these traits showed weaker responses in warmer regions, indicating greater sensitivity to environmental changes under cold conditions [66]. Plants in cold environments generally evolve smaller, thicker, and denser leaves to minimize energy loss and freezing risks [67,68]. Prolonged cold selection has led to the accumulation of cold-tolerant functional traits, promoting clear patterns of adaptive evolution [69]. However, ongoing climate warming could disrupt these adaptations, threatening the stability of alpine and high-latitude vegetation. Light availability also significantly shapes leaf functional traits. Our results showed that SLA and LDMC varied significantly in regions with high sunshine durations (>2500 h) (Figure 6a and Figure 9b), while the leaf area varied prominently in regions with low sunshine durations (<1500 h) (Figure 7a). Other studies have demonstrated that plants under high-light conditions develop smaller thicker leaves for radiation resistance, whereas, under low-light conditions, they expand their leaf area to optimize light capture [70,71,72,73]. The functional differences between the tropical canopy and under-story plants exemplify adaptive evolution driven by light gradients. A study conducted in China found that *Pinus koraiensis* seedlings adopt specific leaf resource-allocation strategies in response to temperature and light intensity variations. Specifically, under high-temperature and high-light conditions, the seedlings increased their specific leaf weight, leaf thickness, and tissue density to enhance leaf resistance and structural stability, thereby reducing water loss. Under moderate temperatures, the leaf photosynthetic rate peaked, indicating optimal resource-use efficiency under favorable environmental conditions. In addition, the concentrations of key nutrients such as nitrogen and phosphorus declined with increasing temperatures. These shifts suggest that plants coordinate structural investment and nutrient regulation to adapt to environmental fluctuations, maintaining a balance between growth and survival [74].

Soil heterogeneity is another crucial driver of leaf trait variation. The SLA, leaf area, leaf thickness, and LDMC all exhibited significant differences across various soil types (Figure 6b, Figure 7b, Figure 8b and Figure 9b), which reflects how the soil texture, pH, and particle size influence water and nutrient absorption, thereby shaping plant resource-use strategies [51]. Harsh environmental conditions in high-elevation areas, such as low temperatures, intense radiation, and nutrient scarcity, limit plant growth and biomass accumulation. Plants adjust their leaf functional traits to survive these combined stresses. Our study showed that the SLA and leaf thickness varied significantly at mid-high elevations (1000–3500 m) (Figure 6a and Figure 8a), while leaf area exhibited the most significant variation at extremely high elevations (>3500 m) (Figure 7a). The combined stresses of low temperature, high radiation, and low oxygen availability in alpine environments have driven the evolution of plant functional traits characterized by dwarfism, structural reinforcement, and high resource-use efficiency [75]. With increasing elevation, plants exhibit corresponding changes in their leaf morphology, structure, and physiological function. Other studies have shown that *Quercus guyavifolia* in the Qilian Mountains of the Qinghai–Tibet Plateau adopt resource-conserving leaf allocation strategies along elevational gradients, such as reducing their leaf area. Furthermore, studies on three angiosperm species in the northeastern Qinghai–Tibet Plateau have confirmed that leaf photosynthetic capacity declines significantly with an increasing elevation [76,77]. These studies confirm that alpine trait variation is jointly driven by topographic heterogeneity and environmental stress. Nevertheless, climate-induced upward shifts in alpine vegetation belts may restructure plant functional trait patterns.

Overall, based on a comprehensive meta-analysis, this study revealed that the SLA, leaf area (LA), leaf thickness (LT), and LDMC exhibit significant and heterogeneous responses across environmental gradients, consistent with the global Leaf Economics Spectrum (LES) framework [60]. The Leaf Economics Spectrum (LES) theory posits that plant leaf traits are distributed from resource-acquisition to resource-conservation strategies. At one end of the spectrum is an acquisitive strategy, characterized by a high specific leaf area (SLA), high photosynthetic rates, and short leaf lifespans. In contrast, the other end represents conservative strategies, marked by a low SLA, lower photosynthetic capacity, and extended leaf longevity. This spectrum reflects plant trade-offs and adaptive techniques in response to varying environmental conditions. Water availability, temperature, light, soil properties, and topography jointly drive plant functional strategies. Specifically, drought, cold, and soil impoverishment promote an increased leaf thickness and LDMC, reinforcing conservative resource-use strategies. Meanwhile, plants at mid-high elevations adjust their SLA, LA, and LT to adapt to low temperatures and nutrient limitations. Consistent with findings from drought simulation experiments in temperate grasslands, our results confirm that plants adjust leaf traits to alleviate water stress, supporting the prevalence of conservative functional adaptations [78]. In summary, this study supports the global Leaf Economics Spectrum and elucidates the specific effects of environmental factor variation on plant leaf functional traits across diverse ecological gradients.

### 4.3. Plant Architecture, Leaf Traits and Environmental Stress

Plant species exhibit comprehensive, adaptive responses to environmental pressures through diverse morphological, physiological, and molecular strategies developed under prolonged ecological stress [75]. These adaptations enhance plant tolerance to extreme environments and drive evolutionary processes, enabling the more efficient utilization of limited resources and improved resilience to environmental uncertainties. This study revealed that plant functional trait responses are predominantly observed in extreme environments, including those with extreme temperatures, drought conditions, nutrient-poor soils, and high elevations. Under such stressful conditions, plant populations experience stronger pressures for survival, which leads to the adjustment of their functional traits [79,80]. This diversification of traits expands the ecological niche breadth, enhances population stability, and improves ecosystem adaptability, reflecting the niche differentiation and diversity evolution at the community level [81,82].

Drought stress profoundly impacts plant’s physiological, ecological, and genetic characteristics. Our results showed that in regions with low precipitation and soil moisture, traits such as the stem diameter, SLA, leaf thickness, and root-to-shoot ratio were susceptible to environmental changes (Figure 4a, Figure 5b, Figure 6b and Figure 8a). Studies on drought stress frameworks suggest that drought facilitates competitive plant interactions [83]. Research on *Leymus chinensis* in the Inner Mongolia Plateau similarly indicates a high sensitivity of plant functional traits to extreme drought conditions [84]. Under severe drought, plants tend to shift from a “tolerant” to an “avoidant” strategy, dynamically adjusting traits such as their leaf water content and SLA [85].

In regions with short photo periods, plants require more extended periods to accumulate sufficient light for growth, making them more sensitive to changes in the sunshine duration [86]. Our findings show that plant height and leaf area were significantly affected by environmental changes in areas with low sunshine durations (Figure 3a and Figure 7a). A study on the Qinghai–Tibetan Plateau also demonstrated that the alpine grassland phenology is highly sensitive to climate change, with plant growth and development closely responding to variations in temperature and precipitation due to limited sunshine [87].

Plants growing at high elevations, exposed to extreme conditions such as low temperatures, drought, and intense ultraviolet radiation, have evolved various adaptive strategies, including phenotypic plasticity [88]. Our study showed that environmental changes at mid to high-elevation regions significantly affected the plant height, leaf area, SLA, leaf thickness, and root-to-shoot ratio (Figure 3a, Figure 5a, Figure 6a, Figure 7a and Figure 8a). Similar conclusions were drawn from experimental studies on *Saussurea* species where the adaptive changes helped to cope with extreme high-altitude conditions [89] and from research in Slovenia, demonstrating the influence of UV radiation and temperature variability on alpine plant functional traits [90]. A comparative analysis was conducted on the leaf traits of *Cotinus szechuanensis* along an elevational gradient in the upper Minjiang River region of China. The results indicated that with an increasing elevation, both the total leaf thickness and palisade tissue thickness of *Cotinus szechuanensis* exhibited an increasing trend [91]. Under the extreme environmental stresses of the alpine periglacial zones of the Qinghai–Tibet Plateau, species have evolved various specialized structures, including cushion plants, woolly plants, and nodding plants [92]. High-altitude plants typically develop traits such as antifreeze protein accumulation, small and thickened leaves, and enhanced root functionality to survive harsh environments [69].

In soils with low organic matter, plant functional traits exhibit high sensitivity to environmental changes [93]. Our findings showed that the plant height, DBH, and root-to-shoot ratio were significantly affected by environmental changes under low-soil-organic-matter conditions (Figure 3a, Figure 4b and Figure 5a). Previous studies have shown that soils with low organic matter contents exhibit heightened sensitivity to temperature and moisture fluctuations [94]. Empirical evidence also indicates that different soil conditions can decouple above-ground and below-ground plant trait responses, particularly under low-organic-matter conditions [95].

In the widely distributed saline–alkaline soils of China, the organic matter content is generally low. Poor soil aggregation and low microbial activity lead to reduced water retention and nutrient supply capacity, severely constraining normal plant growth and ecosystem functioning. Thus, the deficiency of soil organic matter significantly weakens plant stress tolerance and growth capacity, constituting a major ecological stress factor that limits plant survival [96].

The organic matter content plays a critical role in supporting plant growth by maintaining the soil water-holding capacity and nutrient availability. Soils with low organic matter may reduce water retention and nutrient supply, making plants more vulnerable to drought or temperature extremes [97].

## 5. Conclusions

As global warming and the frequency of extreme climate events intensify, plant habitats are becoming increasingly complex, necessitating in-depth and systematic investigations into plant response mechanisms to environmental change. This study presents the first China-scale trait-environment response database, integrating data from 115 field-based studies and 849 effect sizes. By combining architectural and leaf functional traits, we established a multi-factor, multi-trait, and multi-gradient integrative analytical framework. Using a combined approach of meta-analysis and random forest modeling, we comprehensively assessed the effects of 11 environmental factors on 7 categories of plant traits. Results revealed that hydrothermal conditions and soil properties are the primary drivers of structural and functional trait variation. In extreme environments such as arid, nutrient-poor, and high-altitude regions, plants exhibited pronounced adaptive adjustments. This study systematically elucidates the coordinated variation patterns of the plant height, diameter at breast height (DBH), root-to-shoot ratio, leaf area, specific leaf area (SLA), leaf thickness, and leaf dry matter content in response to environmental change across China. These findings offer a robust theoretical foundation for understanding the ecological adaptation strategies of Chinese plants across diverse environmental gradients under climate change. Moreover, the analytical framework and methodology developed in this study provide a transferable model for investigating plant responses to climate change at a global scale. The study also delivers strong scientific evidence and decision-making support for ecosystem management and biodiversity conservation practices.

Although previous studies have addressed plant responses to environmental change, further progress is needed in several key areas: strengthening long-term dynamic monitoring to explain the spatiotemporal mechanisms of trait variation, systematically uncovering functional coordination among plant organs, and constructing standardized trait databases; developing predictive frameworks that integrate physiological and climate models. While this study presents a comprehensive analysis based on data from China, it remains limited by the regional scope, insufficient identification of interaction effects, and restricted spatiotemporal coverage. Future research should aim to expand and refine these findings through the integration of global datasets and long-term observational networks.

## Figures and Tables

**Table 1:** Data classification and grouping.

Categorical Variables	Subgroups			
Elevation [m]	0–1000	1000–3500	>3500	
Plant Functional Group (PFG)	Tree	Shrub	Herbaceous Plant	
Mean Annual Temperature [°C]	0–5	5–15	>15	
Mean Annual Precipitation [mm]	0–500	500–1000	1000–2000	
Scale	Big	Small		
Soil Organic Matter [g/kg^−1^]	0–10	10–20	20–30	>30
Soil Moisture Content [%]	0–10	10–20	20–30	>30
Mean Annual Sunshine Hours [h]	0–1500	1500–2500	>2500	
Relative Humidity [%]	0–60	60–100		
Climate	Temperate Continental Climate	Temperate Monsoon Climate	Subtropical Monsoon Climate	Plateau Climate
Soil type	Desert Soil Chestnut Soil Paddy Soil	Red Soil Fluvo-aquic Soil Lithosol	Brown Soil Meadow Soil Alpine Soil	Black Soil Loess

## Data Availability

Data will be provided upon request.

## Supplementary Materials

The following supporting information can be downloaded at: https://www.mdpi.com/article/10.3390/plants14111717/s1. Supplementary Materials S1: A correspondence table between soil types in the Chinese Soil Classification System and their approximate equivalents in the World Reference Base for Soil Resources (WRB); Supplementary Materials S2: A list of references used for the meta-analysis in this study; Supplementary Materials S3: A list of plant species involved in the research.
