# Supplementary material for: Plant Architectural Structure and Leaf Trait Responses to Environmental Change: A Meta-Analysis
Source: Plants (Basel). 2025 Jun 4;14(11):1717. doi: 10.3390/plants14111717 (PMC12157246; doi:10.3390/plants14111717)
Supplement: Supplementary file 1 [file plants-14-01717-s001.zip › Supplementary S1 Comparative Table of Chinese Soil Classification System and International Soil Classification System.pdf]

Chinese Soil Types and Their Approximate WRB Equivalents

Chinese Soil Type	Approximate WRB Equivalent	Notes
Desert Soil	Arenosols / Calcisols / Gypsisols	Depending on parent material—sandy, calcareous, or gypsic soils common in arid regions
Red Soil	Acrisols / Ferralsols	Strongly weathered, acidic, iron- and aluminum-rich soils in subtropical to tropical climates
Brown Soil	Cambisols / Luvisols	Moderately developed soils, typical of temperate forest zones
Black Soil	Chernozems / Phaeozems	Rich in organic matter, commonly found in steppe and prairie regions
Chestnut Soil	Kastanozems	Semi-arid steppe soils with less organic matter than Chernozems
Fluvo-aquic Soil	Fluvisols	Young soils developed from recent river alluvium
Meadow Soil	Gleysols / Stagnosols	Soils in moist or periodically waterlogged conditions
Paddy Soil	Anthrosols (Hydragric)	Human-modified soils under long-term paddy rice cultivation
Lithosol	Leptosols	Shallow, weakly developed soils over hard rock
Alpine Soil	Cryosols	Cold-region soils, often influenced by permafrost or seasonal freezing
Loess (Loessial Soil)	Calcisols / Cambisols	Derived from loess deposits, classification depends on soil development stage
