# Supplementary material for: Plant Architectural Structure and Leaf Trait Responses to Environmental Change: A Meta-Analysis
Source: Plants (Basel). 2025 Jun 4;14(11):1717. doi: 10.3390/plants14111717 (PMC12157246; doi:10.3390/plants14111717)
Supplement: Supplementary file 1 [file plants-14-01717-s001.zip › Supplementary S2 Literature used in the study.pdf]

1. Xu, Y.K. Response of twigs functional traits and axillary buds biochemical characteristics of Prunus persica Batsch. Var. Duplex Rehd to habitat changes. Master's Thesis, Sichuan Agricultural University, Ya'an, China, 2022. (In Chinese)
2. Zhu, R.Q.; Liu, M.L.; Li, G.; Kang, H.M.; Yang, T.; Wang, Z.Y. Responses of Leaf Functional Traits of Reaumuria soongorica in Two Different Desert Habitats. J. Northwest For. Univ. 2020, 35, 29–34. (In Chinese)
3. Zhang, Q.; Lü, G.; Tian, X. Characteristics of organ biomass allocation of Cenchrus longispinus in four different habitats. Plant Quarantine 2022, 36 (4), 33–41. https://doi.org/10.19662/j.cnki.issn1005-2755.2022.00.012.(In Chinese)
4. Ilyas, M.; Liu, Y.Y.; Shah, S.; Ali, A.; Khan, A.H.; Zaman, F.; Zhang, Y.C.; Saud, S.; Adnan, M.; Ahmed, N.; et al. Adaptation of functional traits and their plasticity of three ornamental trees growing in urban environment. Sci. Hortic. 2021, 286, 110248. https://doi.org/10.1016/j.scienta.2021.110248.
5. Wu, X.; Zheng, X.J.; Mu, X.H.; Li, Y. Differences in Allometric Relationship of Two Dominant Woody Species Among Various Terrains in a Desert Region of Central Asia. Front. Plant Sci. 2021, 12, 754887. https://doi.org/10.3389/fpls.2021.754887.
6. Tayir, M.; Dai, Y.; Shi, Q.; Abdureyim, A.; Erkin, F.; Huang, W. Distinct Leaf Functional Traits of Tamarix chinensis at Different Habitats in the Hinterland of the Taklimakan Desert. Front. Plant Sci. 2023, 13, 1094049. https://doi.org/10.3389/fpls.2022.1094049.
7. Liu, Y.; Li, Z.; Xu, L.; Fu, Q.; Wang, Y. Effects of Region and Elevation on Adaptation of Leaf Functional Traits of an Invasive Plant Erigeron annuus in China. Phyton 2022, 91, 115–128. https://doi.org/10.32604/phyton.2022.015395.
8. Qiu, J.; Han, A.; He, C.; Dai, X.; Jia, S.; Luo, Y.; Hao, Z.; Yin, Q. Functional Traits of Quercus aliena var. acuteserrata in Qinling Huangguan Forest Dynamics Plot: The Relative Importance of Plant Size and Habitat. Forests 2022, 13, 899. https://doi.org/10.3390/f13060899.
9. Wang, Y.; Zhang, Y.; Mao, K.; Li, W.; Cheng, X. Morphological Responses of Bombax ceiba to Habitat Heterogeneity in Southwest China. Front. Ecol. Evol. 2023, 10, 1118045. https://doi.org/10.3389/fevo.2022.1118045.
10. Zheng, B.-Q.; Zou, L.-H.; Li, K.; Wan, X.; Wang, Y. Photosynthetic, Morphological, and Reproductive Variations in Cypripedium Tibeticum in Relation to Different Light Regimes in a Subalpine Forest. PLoS ONE 2017, 12, e0181274, doi:10.1371/journal.pone.0181274.
11. Hu, X.; Zhang, W.; Zhou, J. Plastic Responses in Tree Architecture to Different Light Intensity Habitats: A Case of Chinese Cork Oak. Polish Journal of Ecology 2016, 64, 500–508, doi:10.3161/15052249PJE2016.64.4.005.
12. Hu, D.; Lv, G.; Qie, Y.; Wang, H.; Yang, F.; Jiang, L. Response of Morphological Characters and Photosynthetic Characteristics of Haloxylon Ammodendron to Water and Salt Stress. Sustainability 2021, 13, 388, doi:10.3390/su13010388.
13. Zhang, M.; Ma, K.; Liu, T.; Tang, L.; Khan, A.A.; Yang, T.; Zheng, P.; Wang, T.; Han, J.; Shi, F. Responses in Phenotypic Plasticity of Amaranthus palmeri and Polygonum orientale to Soil Factors under Different Habitats. Clean–Soil Air Water 2020, 48, 1900203. https://doi.org/10.1002/clen.201900203.
14. Wang, C.; Lu, X.; Yang, T.; Zheng, Y.; Chen, L.; Liu, L.; Ni, J. Should More Individuals Be Sampled When Measuring Functional Traits of Tree Species in Habitat-Heterogeneous Karst Forests? Front. Ecol. Evol. 2023, 11, 1175031. https://doi.org/10.3389/fevo.2023.1175031.
15. Yang, S.J.; Sun, M.; Zhang, Y.J.; Cochard, H.; Cao, K.F. Strong leaf morphological, anatomical, and physiological responses of a subtropical woody bamboo to contrasting light environments. Plant Ecol. 2014, 215, 97–109. https://doi.org/10.1007/s11258-013-0281-z.
16. Li, Q.; Zhao, C.Z.; Kang, M.P.; Li, X.Y. The Relationship of the Main Root–Shoot Morphological Characteristics and Biomass Allocation of Saussurea salsa under Different Habitat Conditions in

Sugan Lake Wetland on the Northern Margin of the Qinghai-Tibet Plateau. Ecol. Indic. 2021, 128, 107836. https://doi.org/10.1016/j.ecolind.2021.107836.
17. Yan, R.L. Genetic Diversity Analysis of Salsola passerina Populations under Different Habitats in Alxa Plateau. Master’s Thesis, Inner Mongolia University, Hohhot, China, 2020. (In Chinese)
18. Qiao, Y.Q. Genetic Diversity Analysis of Reaumuria soongorica Populations under Different Habitats in Alxa Desert Area. Master’s Thesis, Inner Mongolia University, Hohhot, China, 2019. (In Chinese)
19. Ren, P.; Yang, H.; Tang, Y.; et al. Phenotypic Plasticity of Sophora moorcroftiana in Response to Soil Moisture. J. Minzu Univ. China (Nat. Sci. Ed.) 2024, 33 (01), 36–45. (In Chinese)
20. Pan, Y.L. Functional Traits and Environmental Adaptation Strategies of Kandelia candel in Coastal Wetlands. Master’s Thesis, Fujian Agriculture and Forestry University, Fuzhou, China, 2018. (In Chinese)
21. Qiang, S.B.; Song, Y.; Zhou, R.L. Morphological Plasticity and Environmental Adaptation Characteristics of Leymus mollis along Coastal Gradients. J. Ludong Univ. (Nat. Sci. Ed.) 2020, 36, 224–232. (In Chinese)
22. Zhou, H.Y.; Wang, J.N.; Fu, X.Q.; Xiang, S.; Wang, Y.X.; Gao, J.; Wu, Y. Effects of Different Light Environments on Phenotypic Plasticity of Three Shrubs in Southern China Grasslands. Chin. J. Appl. Environ. Biol. 2014, 20, 962–970. (In Chinese)
23. Liu, W.L.; Xu, D.M.; Shi, J.M.; Xu, A.Y. Changes in Cluster Structure and Leaf Functional Traits of Agropyron mongolicum Populations across Different Plant Communities. Acta Pratacult. Sin. 2022, 31, 72–80. (In Chinese)
24. Li, Y.; Cui, J.; Su, Y. Specific leaf area and leaf dry matter content of some plants in different dune habitats. Acta Ecol. Sin. 2005, 25 (2), 304–311.(In Chinese)
25. Zhang, Y.G.; Han, M.; Han, Z.M.; Liu, C.J.; Yang, L.M. Growth and photosynthetic characteristics of Epimedium koreanum Nakai in different habitats. Acta Ecol. Sin. 2012, 32, 1442–1449. (In Chinese)
26. Zuo, C.; Wang, M.; Wang, L.; et al. Trade-Off Characteristics of Biomass Allocation in Crotalaria pallida under Different Habitats. Acta Agrestia Sin. 2020, 28 (02), 454–460. (In Chinese)
27. Chen, M.Y. Effects of Different Habitats on the Architecture of Landscape Plants. Master’s Thesis, Fujian Agriculture and Forestry University, Fuzhou, China, 2015. (In Chinese)
28. Ma, Y.; Zang, G.; Zhao, J.; et al. Clonal Architecture and Biomass Characteristics of Cynodon dactylon Populations in Different Habitats. J. Trop. Crops 2019, 40 (08), 1495–1500. (In Chinese)
29. Jiang, R.; Guo, C.B.; Pan, L.L.; Wu, D.R.; Wang, H.B. Flowering Branch Traits and Flowering Characteristics of Magnolia grandiflora under Different Habitats. J. Shanghai Normal Univ. (Nat. Sci. Ed.) 2023, 52, 453–460. (In Chinese)
30. Xiang, F.; Zhou, Q.; Tian, X.R.; Chen, G.X.; Xiao, Y. Leaf Morphology and PSII Chlorophyll Fluorescence Parameters in Leaves of Sinosenecio jishouensis in Different Habitats. Acta Ecol. Sin. 2014, 34. https://doi.org/10.5846/stxb201210291501. (In Chinese)
31. Zhang, Z.; Wang, W.; Du, K.; et al. Effects of Different Habitat Conditions on the Growth and Development of Elymus nutans. J. Inner Mongolia Agric. Univ. (Nat. Sci. Ed.) 2018, 39 (02), 53–57. DOI:10.16853/j.cnki.1009-3575.2018.02.008. (In Chinese)
32. Jin, Q.; Liu, J. Study on the Influence of Different Habitats for the Growth of Drepanostachyum luodianense. Cent. South For. Inventory Plan. 2014, 33 (04), 52–56+66. DOI:10.16166/j.cnki.cn43-1095.2014.04.015. (In Chinese)
33. Chen, F.R. The Studies On Growth Traits and Photosynthetic characteristics of Polygonatum cyrtonema Hua In different habitat conditions. Master’s Thesis, Fujian Agriculture and Forestry University, Fuzhou, China, 2013. (In Chinese)

34. Dai, Q.; Huang, X.; Huang, X.; et al. Comparison of Growth and Photosynthetic Characteristics of Paeonia ostii under Different Habitat Conditions. J. Southwest Univ. (Nat. Sci. Ed.) 2018, 40 (09), 53–58. DOI:10.13718/j.cnki.xdzk.2018.09.009. (In Chinese)
35. Sun, Y. Growth and Development of Jacaranda mimosifolia in different habitats. Master’s Thesis, Sichuan Agricultural University, 2016. (In Chinese)
36. Xie, H.; Tao, L.; Yang, W.; et al. A Comparative Study on Leaf Traits and Anatomical Structures of ‘Gold Delicious’ Apple Collected from Different Habitats. Chin. Agric. Sci. Bull. 2017, 33 (06), 91–96. (In Chinese)
37. Zhong, J.; Xu, Y.; Zeng, F.; et al. Analysis on the growth characteristics of Eupatorium catarium in different types of habitats. Guangxi Plant 2014, 34 (01), 68–73+76. (In Chinese)
38. Tang, W. The adaptability of Alternanthera philoxeroides grown in different habitats with contrasting water conditions. Master’s Thesis, Jiangxi Science and Technology Normal University, 2020. DOI:10.27751/d.cnki.gjxkj.2020.000280. (In Chinese)
39. Huang, Z.; Yang, L.; Wang, Y.; et al. The characteristics of tree shape structure and the influencing factors of Bombax ceiba L. in different habitats. Chin. J. Ecol. 2022, 41 (08), 1552–1559. DOI:10.13292/j.1000-4890.202208.011. (In Chinese)
40. Wang, Y.; Qi, X.; Yang, H.; et al. Morphological Structure and Biomass Allocation Characteristics of Echinops gmelini under Different Habitats. J. Desert Res. 2018, 38 (04), 756–764. (In Chinese)
41. Liu, L.C.; Du, G.G.; Si, W.J.; Wang, F.; Luo, H.J.; Zhou, Z.J. Phenotypic variation and covariation in natural populations of the exotic weed Gaura parviflora in different habitat. Acta Pratacult. Sin. 2015, 24, 41–51. (In Chinese)
42. Liu, Q.; Liu, Y.Q.; Gu, X.R. Study on the Growth Characteristics of Roadside Trees of Cinnamomum camphora in Different Habitats and Its Response to Snow and Ice Hazards. Chin. Landsc. Archit. 2020, 36, 112–117. https://doi.org/10.19775/j.cla.2020.02.0112. (In Chinese)
43. Li, L.; Sun, Y.T.; Ren, X.; Mo, L.N.; Hou, Y.H.; Yuan, Z.N. Functional Traits of Branches and Leaves, and Their Correlation with Environmental Factors for Lavandula angustifolia Mill. in Different Growth Stages. J. Northeast For. Univ. 2021, 49, 21–28. https://doi.org/10.13759/j.cnki.dlxb.2021.12.010. (In Chinese)
44. Cao, Y.; Xiao, Y.; Zhou, B.; et al. A the phenotypic plasticity of alternanthera philoxeroides to different water habitats. J. Jinggangshan Univ. Nat. Sci. 2012, 33 (3), 102–106.(In Chinese)
45. Zhou, T.; Cui, Y.C.; Ye, Y.Y.; Zhao, W.J.; Hou, Y.J.; Wu, P.; Ding, F.J. Leaf functional traits of typical karst forest plants under different niches. J. Cent. South Univ. For. Technol. 2022, 42, 129–140. https://doi.org/10.14067/j.cnki.1673-923x.2022.10.015. (In Chinese)
46. Tan, L.P.; Zeng, W.Q.; Liu, X.A.; Zhou, X.H.; Yang, X.; Peng, P.H. Differences in Functional Traits among Provenances of Paeonia delavayi and Their Relationship with Environmental Factors. North. Hortic. 2019, 48–55. (In Chinese)
47. Chen, L.R.; Ma, R.H.; Wang, F.; Xia, K.; Deng, P.F.; Xu, X.N. Variations of leaf and fine-root functional Traits of five garden tree species across an urban-rural gradient. Chin. J. Ecol. 2023, 42, 1281–1289. https://doi.org/10.13292/j.1000-4890.202305.027. (In Chinese)
48. Xie, H.J. Studies on the Ecological Adaptability of Golden Delicious Apple in Sichuan and Tibet Plateau. Ph.D. Thesis, Sichuan Agricultural University, Ya’an, China, 2016. (In Chinese)
49. Tan, H.W. Study on southwest Sichuan Fagopyrum cymosum population morphological differences and different habitats impact. Master’s Thesis, Sichuan Agricultural University, Ya'an, China, 2018. (In Chinese)
50. Zhou, J.; Yang, S.; Hua, S.G.; Wu, G.L. Relationship Between Leaf Functional Traits and Soil Nutrients of Pinus dabeshanensis. J. Anqing Norm. Univ. (Nat. Sci. Ed.) 2023, 29, 96–102. https://doi.org/10.13757/j.cnki.cn34-1328/n.2023.02.016. (In Chinese)

51. Ma, J.; Wu, L.F.; Wei, X.; Ye, W.H.; Cao, H.L.; Shen, H. Habitat adaptation of two dominant tree species in a subtropical monsoon forest: leaf functional traits and hydraulic properties. Guangxi Plants 2015, 35, 261–268. (In Chinese)
52. Li, Y.; Yao, J.; Yang, S.; Hou, J.H. Leaf functional traits of main tree species at different environmental gradients in Dongling Mountain, Beijing. J. Beijing For. Univ. 2014, 36, 72–77. https://doi.org/10.13332/j.1000-1522.2014.01.019. (In Chinese)
53. Han, L. The relationships among the leaf traits of Polygonum viviparum in different population densities in Gaihai wetland. Master's Thesis, Northwest Normal University, Lanzhou, China, 2017. (In Chinese)
54. Lü, J. Comparative Study on Leaf Structure and Functional Traits of Five Greening Shrubs in Different Habitats in Nanning, Guangxi. Master's Thesis, Guangxi University, 2023. DOI:10.27034/d.cnki.ggxiu.2023.000634. (In Chinese)
55. Qi, Y.K.Study on the Allometry and Rhizosphere Microorganism Diversity of Different Ecotypes of Phragmites australis in Hexi Corridor. Master's Thesis, Gansu Agricultural University, Lanzhou, China, 2023. (In Chinese)
56. Jiang, S.X.; Zhao, P. Morphological structure and biomass allocation of Agriophyllum squarrosum in different habitats of east section of Hexi Corridor. Agric. Res. Arid Areas 2023, 41, 248–256. (In Chinese)
57. Yue, X.Y.; Yue, W.; Yan, K. Study on the characteristics of rhizospheric microbial communities in the heterogeneous habitats of coastal wetlands and in the Yellow River Delta. J. Northeast For. Univ. 2023, 51, 18–21+30. https://doi.org/10.13759/j.cnki.dlxb.2023.03.019. (In Chinese)
58. Yang, S.R. Adaptation of Functional Traits of Phragmites australis to Different Water Habitats in the Yellow River Delta. Master's Thesis, Shandong University, Jinan, China, 2020. (In Chinese)
59. Liu, X.; Cai, L.; Zhang, Y.; et al. Variations in the stand biomass of Pinus taiwanensis forests along an altitudinal gradient. J. For. Environ. 2023, 43 (5), 516–522. https://doi.org/10.13324/j.cnki.jfcf.2023.05.009.(In Chinese)
60. Tang, Y.; Wen, Z.M.; Liu, J.; Yang, Y.T.; Wang, Y. Adaptation Mechanism of Robinia Pseudoacacia Under Different Site Conditions in Loess Hill and Gully Region. Soil Water Conserv. Bull. 2019, 39, 46–53. https://doi.org/10.13961/j.cnki.stbctb.2019.05.007. (In Chinese)
61. Zhu, X.W. Ecological Adaptation of Some Dominant Plants in Otindag Sandland. Ph.D. Thesis, Graduate School of Chinese Academy of Sciences (Institute of Botany), Beijing, China, 2004. (In Chinese)
62. Zhang, X.; Li, R.X.; Zheng, Z.; Li, Z.H.; Gong, L.; Luo, Y.; Wu, X. Leaf functional traits of Tamarix ramosissima in extremely arid region and their relationship with soil physicochemical factors. Acta Ecol. Sin. 2023, 43, 3699–3708. (In Chinese)
63. Zheng, Y.; Zhang, H.Y.; Zou, H.C.; Huang, T.S.; Bai, Z.Q.; Zhen, X.X. Branch Structure Characteristics of Betula Platyphylla in Different Habitats in Mountainous Forests of Northwest Hebei. Sci. Technol. Eng. 2024, 24, 970–979. (In Chinese)
64. Cao, J.Y. Plant functional traits of twigs and their relationships with environmental factors in beishan mountain of jinhua, zhejiang province. Master's Thesis, Zhejiang Normal University, Jinhua, China, 2020. (In Chinese)
65. Differences and Variations of Functional Traits of Cenchrus pauciflorus in four Habitats in Horqin Sandy Land. Master's Thesis, Inner Mongolia University for Nationalities, Tongliao, China, 2022. (In Chinese)
66. Zhou, Y.; Jiao, L.; Qin, H.J.; Wu, J.J.; Che, X.C. Response of Leaf Functional Traits of Clonal Plant Phragmites australis to Heterogeneous Environments. Chin. J. Appl. Ecol. 2022, 33, 2171–2177. https://doi.org/10.13287/j.1001-9332.202208.010. (In Chinese)

67. Zha, G.D. The space differences in arborvitae function in Platycladus orientalis in Lanzhou northern mountains. Master’s Thesis, Northwest Normal University, Lanzhou, China, 2015. (In Chinese)
68. Zhang, Y.; Tian, Q.; Huang, R.; Lan, R. Responses of typical plant functional traits among summer-flowering tree species in heterogeneous city habitats in Lanzhou City of northwestern China. J. Beijing For. Univ. 2023, 45, 90–99. (In Chinese)
69. Chen, L.; Zhang, M.L.; Li, J.H.; Chen, H.L.; Zhao, J.Y. Comparative Study on Growth Dynamics of Reed Communities in Heterogeneous Habitats in Liaohe River Delta. J. Jiangxi Agric. Univ. 2020, 32, 62–67. https://doi.org/10.19386/j.cnki.jxnyxb.2020.10.12. (In Chinese)
70. Li, Q.; Li, J.P.; Zhao, B.C.; Xu, P.F.; He, M. Growth Traits of Miscanthus sinensis under Different Habitats in Benxi, Liaoniang Province. J. Northeast For. Univ. 2013, 41, 18–21. https://doi.org/10.13759/j.cnki.dlxb.2013.11.005. (In Chinese)
71. Zhao, X.L. The clonal growth of Hedysarum mongolicum and its water physiological effects under different habitats in the Mu Us Sandy Land. Master’s Thesis, Ningxia University, Yinchuan, China, 2021. (In Chinese)
72. Liu, T.; Tang, Y.Y.; Yang, H.Y.; Li, L.; Shi, S. Response of Functional Traits of Ammopiptanthus mongolicus to Soil Moisture. J. Minzu Univ. China (Nat. Sci. Ed.) 2024, 33, 28–35. (In Chinese)
73. Li, D.L.; Liu, S.Z.; Ji, Y.F.; Li, C.L.; Zhang, Z.P.; Tang, W.D.; Chai, C.W.; Jiang, S.X.; Wu, H. Community Structure and Species Diversity of Atraphaxis bracteata In Minqin Liangucheng Nature Reserve. J. Northwest For. Univ. 2016, 31, 85–89+181. (In Chinese)
74. Wang, Q. Leaf Trait Characteristics of 110 Garden Greening Plant Species in Nanchang. Master’s Thesis, Jiangxi Normal University, Nanchang, China, 2020. (In Chinese)
75. Jiao, L.; Guan, X.; Liu, X.R.; Dong, X.G.; Li, F. Leaf Functional Traits of Phragmites australis in Inland River Wetlands and Their Responses to Soil Environmental Factors. Arid Zone Res. 2020, 37, 202–211. https://doi.org/10.13866/j.azr.2020.01.23. (In Chinese)
76. Comparisons of Leymus chinensis Characters in Different Habitats in the Inner Mongolian Plateau. Chin. J. Ecol. 1996, 61–64+68. (In Chinese)
77. Liao, M.J.; Wang, Q.B.; Song, M.H.; Dong, M. Clonal Architecture and Ramet Population Characteristics of Leymus chinensis in Different Habitats of the Xilin River Basin, Inner Mongolia. Acta Phytoecol. Sin. 2002, 33–38. (In Chinese)
78. Li, L.A. Response of Effective Components and Growth Physiological Characteristics of Periploca sepium to Ecological Factors in Different Habitats in Ningxia. Master’s Thesis, Ningxia University, Yinchuan, China, 2023. (In Chinese)
79. Zeng, J.J.; Xiao, Y.A.; Zhou, X.J. Clonal Propagation and Community Characteristics of the Invasive Plant Coreopsis lanceolata. Plant Resour. Environ. 2012, 32, 147–150. (In Chinese)
80. Guo, Q.C. Functional Traits Variation Regularity of Invasive Plant Gaura parviflora. Master’s Thesis, Henan University of Science and Technology, Luoyang, China, 2018. (In Chinese)
81. Qiu, X.; Xu, Z.; Tu, Y.; et al. Module Biomass and Allocation Characteristics of Population Components of the Invasive Plant Tagetes minuta in Different Habitats. Guangxi Plant 2021, 41 (03), 447–455. (In Chinese)
82. Qiu, T.; Shang, H.Z.; Huang, X.Q.; et al. Analysis of Growth-Related Traits of the Invasive Species Cenchrus incertus under Heterogeneous Habitats. Mol. Plant Breed. [Online First], 1–10 [2025-03-23]. http://kns.cnki.net/kcms/detail/46.1068.S.20220324.0932.002.html. (In Chinese)
83. Li, X.L.; Wen, H.R.; Wang, X.S.; Yang, J.; Huang, C.M. Phenotypic Plasticity of Distylium chinense Leaves in Relation to Soil Environmental Factors in Heterogeneous Habitats in the Three Gorges Reservoir Region. Acta Ecol. Sin. 2018, 38. https://doi.org/10.5846/stxb201703030353. (In Chinese)
84. Han, X.L.; Zhao, M.S.; Wang, Z.Y.; Ye, L.F.; Lu, S.T.; Chen, S.; Li, Y.; Xie, J.B. Adaptation of Xylem Structure and Function of Three Gymnosperms to Different Habitats. Acta Phytoecol. Sin. 2022, 46, 440–450. (In Chinese)

85. Wang, H.J.; Li, J.R.; Zheng, W.L.; Chen, K.; Chen, W.S.; Ding, H.H. Distribution Pattern and Functional Diversity of Woody Plants in Abies Communities Along Environmental Gradients in the Sejila Mountains. Green Sci. Technol. 2022, 24, 19–22+28. https://doi.org/10.16663/j.cnki.lskj.2022.08.009. (In Chinese)
86. Wang, F.; Chen, W.Y.; Guo, S.J.; Yang, F.; Wang, Q.Q.; Yang, Z.H. Response of Leaf Functional Traits of Calligonum mongolicum to Habitat Changes. Acta Bot. Boreali-Occident. Sin. 2024, 44, 77–87. (In Chinese)
87. Chen, L.R.; Chen, W.Y.; Li, Y.Y.; Chen, J.C. Plasticity of Morphological and Physiological Traits in Caragana korshinskii under Different Habitats of the Water-Wind Erosion Crisscross Region of the Loess Plateau. Acta Bot. Boreali-Occident. Sin. 2016, 36, 573–578. (In Chinese)
88. Han, W.J. Study of Pinus tabulaeformis Plantation Community Characteristics and Regeneration in Different Habitats of Huanglong Mountain Region, Shaanxi. Master's Thesis, Northwest A&F University, Yangling, China, 2013. (In Chinese)
89. Yang, N.; Liu, X.; Zhao, X.; et al. Impacts of Varying Habitats on the External Morphological Structure of Ageratina adenophora Leaves. J. Agric. Sci. 2023, 13 (09), 46–53. (In Chinese)
90. Liang, K.; Fan, Y.; Bu, W.S.; Wang, L.F.; Shi, J.M. Phenotypic Plasticity of A Dominant Bamboo Species(Phyllostachys glauca) in Limestone Mountain in Northwest of Jiangxi Province. J. Jiangxi Agric. Univ. 2017, 39, 1178–1186. https://doi.org/10.13836/j.jjau.2017153. (In Chinese)
91. Qiu, T. Study on Phenotypic Variation and Molecular Ecology of Phragmites australis in Heterogeneous Habitats of the Songnen Plain. Ph.D. Thesis, Northeast Normal University, Changchun, China, 2017. (In Chinese)
92. Yan, D.F.; Yang, Y.F.; Zhao, M.Q. Phenotypic Plasticity of Phragmites australis Population Leaves in Heterogeneous Habitats of the Songnen Meadow. J. Northeast Normal Univ. (Nat. Sci. Ed.) 2012, 44, 78–83. https://doi.org/10.16163/j.cnki.22-1123/n.2012.02.030. (In Chinese)
93. Guo, L. Adaptive Study on Leaf Functional Traits of Nitraria tangutorum in Different Habitats. Master's Thesis, Inner Mongolia Agricultural University, Hohhot, China, 2022. (In Chinese)
94. Shang, H. Morphological and Physiological Variation and Seed Germination Physiology of Dysosma versipellis. Master's Thesis, Northwest University, 2009. (In Chinese)
95. Zhang, X.H. Study on Population Distribution, Morphological Characteristics and Physiological-Biochemical Characteristics of Dactylis glomerata in East and West Sections of Tianshan Mountain North Slope. Ph.D. Thesis, Xinjiang Agricultural University, Urumqi, China, 2015. (In Chinese)
96. Cao, J.J.; Wang, R.; Li, Y.G.; Zhang, G.F.; Guo, J.Y.; Wan, F.H. The Phenotypic Variation and Environmental Adaptability among Different Geographical Populations of Amaranthus palmeri in China. Plant Quarantine 2020, 34, 25–31. https://doi.org/10.19662/j.cnki.issn1005-2755.2020.00.007. (In Chinese)
97. Yuan, Y.; Liu, J.; Gao, X.; et al. Root Traits of Seven Stipa Species and Their Relations with Environmental Factors in Temperate Grasslands. Acta Ecol. Sin. 2022, 42 (21), 8784–8794.(In Chinese)
98. Zhou, T. Stoichiometric Characteristics and Adaptation Mechanisms of Typical Karst Forest Plants in Microhabitats. Master's Thesis, Guizhou University, 2022. DOI:10.27047/d.cnki.ggudu.2022.001561. (In Chinese)
99. Xu, G.F.; Shen, S.C.; Zhang, F.D. Adaptability and Reproductive Characteristics of the Invasive Plant Mikania micrantha under Heterogeneous Environments. Ecol. Environ. Sci. 2014, 23, 1258–1264. https://doi.org/10.16258/j.cnki.1674-5906.2014.08.001. (In Chinese)
100. Dong, H.P.; Cao, H.J.; Xie, L.H.; Huang, Q.Y.; Yang, L.B.; Ni, H.W.; Wang, J.F. Effects of Water Levels in Heterogeneous Habitats on Sexual Reproductive Allocation of Deyeuxia angustifolia. Chin. J. Appl. Ecol. 2022, 33, 378–384. https://doi.org/10.13287/j.1001-9332.202202.004. (In Chinese)

101. Zhang, Y. Characteristics and Assessments of Functional Traits and Anticipated Performances among Urban Summer-Flowering Tree Species in Heterogeneous Habitats. Master's Thesis, Gansu Agricultural University, Lanzhou, China, 2022. (In Chinese)
102. Guan, S.K.; Tao, D.Y.; Zhou, J.Y.; Yan, H.X.; Song, Q.; Luo, S.M. Population Characteristics and Growth Status of Impatiens psittacina in Heterogeneous Habitats. Southwest China J. Agric. Sci. 2022, 53, 2205–2214. (In Chinese)
103. Wei, J. Response and Adaptability of Growth, Leaf Color and Photosynthetic Characteristics to Heterogeneous Habitats of Introduced Acer palmatum. Master's Thesis, Southwest University, Chongqing, China, 2023. (In Chinese)
104. Zhu, L.J.; Wang, S.M.; Xia, J.; et al. Clonal Architecture and Ramet Population Characteristics of Stipagrostis pennata in Different Environments. Arid Zone Res. 2012, 29, 770–775. https://doi.org/10.13866/j.azr.2012.05.01. (In Chinese)
105. Jiao, D.Z.; Rong, Z.; Cao, R.; Yan, Q.Y.; Jiang, Q.X.; Yang, Y.F. Quantitative Characteristics of the Ramet Module of Phragmites australis Populations in Heterogeneous Habitats in the Zhalong Wetland National Reserve. Acta Ecol. Sin. 2017, 37, 7843–7853. (In Chinese)
106. Jiao, D.Z.; Zhong, L.P.; Wang, J.J.; et al. Growth Analysis of the Ramets of Phragmites australis in Different Habitats in Zhalong Wetland. Pratacult. Sci. 2021, 38, 1950–1957. (In Chinese)
107. Jiao, D.Z.; Zhong, L.P.; Yang, J.X.; Yu, B.G. Variation in Functional Traits of Phragmites australis and Their Response to Soil Factors in Different Habitats of Zhalong Wetland. Acta Ecol. Sin. 2023, 43, 9305–9313. https://doi.org/10.20103/j.stxb.202208202387. (In Chinese)
108. Zhang, W.T. The Clonal Architecture of Typha orientalis under Different Water Levels in Marsh Wetlands. Master's Thesis, Northwest Normal University, Lanzhou, China, 2018. (In Chinese)
109. Chen, S.Q. Interaction Between Plant Leaf Functional Traits and Atmospheric Particle Retention. Master's Thesis, Hangzhou Normal University, Hangzhou, China, 2023. (In Chinese)
110. Zhou, L.Q. Study on the Functional Traits of Common Woody Plants in Chongqing Mountain Park. Master's Thesis, Southwest University, Chongqing, China, 2022. (In Chinese)
111. Zhao, Y.Y.; Wang, H.Y. Growth Characteristics of Landscape Trees in Chongqing and Their Response to Habitat. J. Southwest Univ. (Nat. Sci. Ed.) 2019, 41, 7–18. https://doi.org/10.13718/j.cnki.xdzk.2019.11.002. (In Chinese)
112. Zhang, L.M.; Tan, X.; Dong, Z.; Zheng, J.; Yuan, Z.X.; Li, C.X. The Relationship between Plant Functional Traits and Soil Physicochemical Properties in the Riparian Zones of Downtown Chongqing. Acta Ecol. Sin. 2023, 43, 1892–1901. (In Chinese)
113. Wang, H.F.; Mu, L.Q.; Wang, H.C.; Wang, Z.X.; Li, W.X. Comparison of the Growth of Tilia amurensis in Natural and Urban Environments. J. Northwest For. Univ. 2012, 27, 101–105. (In Chinese)
114. Fang, J.; Ye, L.F.; Chen, S.; Lu, S.T.; Pan, T.T.; Xie, J.B.; Li, Y.; Wang, Z.Y. Differences in Anatomical Structure and Hydraulic Function of Xylem in Branches of Angiosperms in Field and Garden Habitats. Acta Phytoecol. Sin. 2021, 45, 650–658. (In Chinese)
115. Yang, F. Divergent Adaptative Mechanism of Modules Functional Traits of Betula Platyphylla in Heterogeneous Habitats in Wudalianchi Volcanoes. Ph.D. Thesis, Northeast Forestry University, Harbin, China, 2021. (In Chinese)
