# Supplementary material for: Plant Architectural Structure and Leaf Trait Responses to Environmental Change: A Meta-Analysis
Source: Plants (Basel). 2025 Jun 4;14(11):1717. doi: 10.3390/plants14111717 (PMC12157246; doi:10.3390/plants14111717)
Supplement: Supplementary file 1 [file plants-14-01717-s001.zip › Supplementary S3 List of species.pdf]

Corresponds to the document number in Annex 2	Article	Species
1	Response of twigs functional traits and axillary buds biochemical characteristics of *Prunus persica Batsch. Var. Duplex* Rehd to habitat changes	*Prunus persica cv．duplex*
2	Responses of Leaf Functional Traits of *Reaumuria soongorica* in Two Different Desert Habitats	*Reaumuria soongorica*
3	Characteristics of organ biomass allocation of *Cenchrus longispinus* in four different habitats	*Cenchrus longispinus*
4	Adaptation of functional traits and their plasticity of three ornamental trees growing in urban environment	*Koelreuteria paniculata*、*Ginkgo biloba*、*Acer truncatum*
5	Differences in Allometric Relationship of Two Dominant Woody Species Among Various Terrains in a Desert Region of Central Asia	*Haloxylon ammodendron*、*Haloxylon persicum*
6	Distinct leaf functional traits of *Tamarix chinensis* at different habitats in the hinterland of the Taklimakan desert	*Tamarix chinensis*
7	Effects of Region and Elevation on Adaptation of Leaf Functional Traits of an Invasive Plant *Erigeron annuus* in China	*Erigeron annuus*
8	Functional Traits of *Quercus aliena var. acuteserrata* in Qinling Huangguan Forest Dynamics Plot：The Relative Importance of Plant Size and Habitat	*Quercus aliena var. acuteserrata*
9	Morphological Responses of *Bombax ceiba* to Habitat Heterogeneity in Southwest China	*Bombax ceiba*
10	Photosynthetic, morphological, and reproductive variations in *Cypripedium tibeticum* in relation to different light regimes in a	*Cypripedium tibeticum*

	subalpine forest	
11	Plastic Responses in Tree Architecture to Different Light Intensity Habitats: A Case of Chinese Cork Oak	*Quercus variabilis*
12	Response of Morphological Characters and Photosynthetic Characteristics of *Haloxylon ammodendron* to Water and Salt Stress	*Haloxylon ammodendron*
13	Responses in Phenotypic Plasticity of *Amaranthus palmeri* and *Polygonum orientale* to Soil Factors under Different Habitats	*Amaranthus palmeri*、*Polygonum orientale*
14	Should more individuals be sampled when measuring functional traits of tree species in habitat-heterogeneous karst forests	*Clausena dunniana* *Platycarya strobilacea*
15	Strong leaf morphological, anatomical, and physiological responses of a subtropical woody bamboo to contrasting light environments	*Sinarundinaria nitida*
16	The relationship of the main root-shoot morphological characteristics and biomass allocation of *Saussurea salsa* under different habitat conditions	*Saussurea salsa*
17	Genetic Diversity Analysis of *Salsola passerina* Populations under Different Habitats in Alxa Plateau	*Salsola passerina*
18	Genetic Diversity Analysis of *Reaumuria soongorica* Populations under Different Habitats in Alxa Desert Area	*Reaumuria soongorica*
19	Phenotypic Plasticity of *Zygophyllum xanthoxylum* in Response to Soil Moisture	*Zygophyllum xanthoxylum*
20	Functional Traits and Environmental Adaptation Strategies of Kandelia candel in Coastal Wetlands	*Kandelia candel*

21	Morphological Plasticity and Environmental Adaptation Characteristics of Leymus mollis along Coastal Gradients	*Leymus mollis*
22	Effects of Light Environments on the Phenotypic Plasticity of Three Shrubs in Southern China Grasslands	*Salix etosia, Rubus setchuenensis, Hydrangea aspera*
23	Changes in Cluster Structure and Leaf Functional Traits of *Agropyron mongolicum* Populations across Different Plant Communities	*Agropyron mongolicum*
24	Specific leaf area and leaf dry matter content of some plants in different dune habitats	*Chenopodium acuminatum*、*Corispermum elongatum*、*Cynanchum thesioides*、*Euphorbia esula*、*Euphorbia humifusa*、*Ixeris chinensis*、*Salsola collina*、*Setaria viridis*、*Astragalus adsurgens*、*Artemisia halodendron*、*Pennisetum centrasiatum*、*Phragmites australis*、*Salix gordejevii*
25	Growth and photosynthetic characteristics of *Epimedium koreanum* Nakai in different habitats	*Epimedium koreanum Nakai*
26	Trade-offs in Modular Biomass Allocation of *Crotalaria spectabilis* in Different Habitat	*Crotalaria spectabilis*
27	Effect of Different Habitats on Architecture of Landscape Plants	*Lagerstroemia indica*、*Prunus persica (L.) Batsch f. duplex Rehd.*、*Prunus campanulata*、*Prunus salicina*、*Prunus mume*、*Magnolia ×soulangeana*、*Osmanthus fragrans (Thunb.) Lour. var. semperflorens Hort.*、

		Camellia japonica、*Cassia surattensis*、*Lagerstroemia speciosa*
28	Clone Architecture and Biomass Characteristics of *Cynodon dactylon* Population in Different Habitats	*Cynodon dactylon*
29	Floral branch traits and flowering characteristics of *Magnolia grandiflora* under different habitats	*Magnolia grandiflora*
30	Leaf morphology and PSIIchlorophyll fluorescence parameters in leaves of *Sinosenecio jishouensis* in Different Habitats	*Sinosenecio jishouensis*
31	Effect of habitats to development of *agropyron michnoi*	*agropyron michnoi*
32	Study on the Influence of Different Habitats for the Growth of *Drepanostachyum luodianense*	*Drepanostachyum luodianense*
33	The Studies On Growth Traits and Photosynthetic characteristics of *Polygonatum cyrtonema* Hua In different habitat conditions	*Polygonatum cyrtonema Hua*
34	Comparison of Growth and Photosynthetic Characteristics of *Paeonia ostii* under Different Habitat Conditions	*Paeonia ostii*
35	Growth and Development of *Jacaranda mimosifolia* in different habitats	*Jacaranda mimosifolia*
36	A Comparative Study on Leaf Traits and Anatomical Structures of'Gold Delicious'Apple Collected from Different Habitats	*Malus domestica 'Golden Delicious'*
37	Analysis on the growth characteristics of *Eupatorium catarium* in different types of habitats	*Eupatorium catarium*
38	The adaptability of *Alternanthera philoxeroides* grown in different habitats with contrasting water	*Alternanthera philoxeroides*

	conditions	
39	The characteristics of tree shape structure and the influencing factors of *Bombax ceiba* L． in different habitats．	*Bombax ceiba*
40	Morphological Structure and Biomass Allocation Characteristics of *Echinops gmelin*i under Different Habitats	*Echinops gmelin*
41	Phenotypic variation and covariation in natural populations of the exotic weed *Gaura parviflora* in different habitat	*Gaura parviflora*
42	Study on the Growth Characteristics of Roadside Trees of *Cinnamomum camphora* in Different Habitats and Its Response to Snow and Ice Hazards	*Cinnamomum camphora*
43	Functional Traits of Branches and Leaves，and Their Correlation with Environmental Factors for *Lavandula angustifolia* Mill． in Different Growth Stages	*Lavandula angustifolia*
44	A the phenotypic plasticity of *alternanthera philoxeroides* to different water habitats	*Alternanthera philoxeroides*
45	Leaf functional traits of typical karst forest plants under different niches	*Cyclobalanopsis phanera*、*Acer wangchii*、*Lindera communis*、*Cornus wilsoniana*、*Platycarya strobilacea*、*Zenia insignis*、*Nandina domestica*、*Brassaiopsis glomerulata*、*Miliusa sinensis*、*Murraya exotica*
46	Differences in Functional Traits among Provenances of *Paeonia delavayi* and Their Relationship with Environmental Factors	*Paeonia delavayi*
47	Variations of leaf and fine-root functional Traits of five garden tree species across an urban-rural	*Photinia×fraseri*、*Euonymus japonicus ‘Aurea-marginatus’*、

	gradient．	*Cinnamomum camphora*、*Ligustrum lucidum*、*Osmanthus fragrans*
48	Studies on the Ecological Adaptability of Golden Delicious Apple in Sichuan and Tibet Plateau	*Malus domestica Borkh*
49	Study on southwest Sichuan *Fagopyrum cymosum* population morphological differences and different habitats impact	*Fagopyrum cymosum*
50	Relationship Between Leaf Functional Traits and Soil Nutrients of *Pinus dabeshanensis*	*Pinus dabeshanensis*
51	Habitat adaptation of two dominant tree species in a subtropical monsoon forest: leaf functional traits and hydraulic properties	*Ardisia quinquegona*、*Aidia canthioides*
52	Leaf functional traits of main tree species at different environmental gradients in Dongling Mountain，Beijing	*Quercus liaotungensis*、*Acer pictum*
53	The relationships among the leaf traits of *Polygonum viviparum* in different population densities in Gaihai wetland	*Polygonum viviparum*
54	Leaf structure and functional trait of five greening shrubs in different habitats in nanning city, guangxi	*Apocynaceae Allamanda*、*Moraceae Ficus*、*Verbenaceae Duranta*、*Rubiaceae Ixora*、*Loganiaceae Fagraea*
55	Study on the Allometry and Rhizosphere Microorganism Diversity of Different Ecotypes of *Phragmites australis* in Hexi Corridor	*Phragmites australis*
56	Morphological structure and biomass allocation of	*Agriophyllum squarrosum*

	Agriophyllum squarrosum in different habitats of east section of Hexi Corridor	
57	Study on the characteristics of rhizospheric microbial communities in the heterogeneous habitats of coastal wetlands and in the Yellow River Delta	*Phragmites australis*
58	Adaptation of Functional Traits of *Phragmites australis* to Different Water Habitats in the Yellow River Delta	*Phragmites australis*
59	Variations in the stand biomass of *Pinus taiwanensis* forests along an altitudinal gradient	*Pinus taiwanensis*
60	Adaptation Mechanism of *Robinia Pseudoacacia* Under Different Site Conditions in Loess Hill and Gully Region	*Robinia Pseudoacacia*
61	Ecological Adaptation of Some Dominant Plants in Otindag Sandland	*Hedysarum laeve Maxim.(Leguminosae)*、*Agropyron cristatum (L.) Gaertn*、*Leymus secalinus (Georgi) Tzvelev*、*Caragana microphylla*
62	Leaf functional traits of *Tamarix ramosissima* in extremely arid region and their relationship with soil physicochemical factors	*Tamarix ramosissima*
63	Branch Structure Characteristics of *Betula Platyphylla* in Different Habitats in Mountainous Forests of Northwest Hebei	*Betula Platyphylla*
64	Plant functional traits of twigs and their relationships with environmental factors in beishan mountain of jinhua, zhejiang province	*Pinus taiwanensis*、*Pinus massoniana*、*Cunninghamia lanceolata*、*Cryptomeria fortunei*、*Schima superba*、*Symplocos sumuntia* 、*Castanopsis sclerophylla*、*Cyclobalanopsis glauca*、*Castanea henryi*、

		Quercus serrata var. Brevipetiolata、*Castanea seguinii*、*Styrax confusus*、*Liquidambar formosana*、*Metasequoia glyptostroboides*、*Dalbergia hupeana*
65	Differences and Variations of Functional Traits of *Cenchrus pauciflorus* in four Habitats in Horqin Sandy Land	*Cenchrus pauciflorus*
66	Responses of leaf functional Traits of clonal plant *Phragmites australis* to heterogeneous environments	*Phragmites australis*
67	The space differences in arborvitae function in *Platycladus orientalis* in Lanzhou northern mountains.	*Platycladus orientalis*
68	Responses of typical plant functional traits among summer-flowering tree species in heterogeneous city habitats in Lanzhou City of northwestern China	*Sophora japonica*、*Koelreuteria paniculata*、*Sambucus williamsii*、*Aesculus chinensis*、*Sorbaria kirilowii*
69	Comparative Study on Growth Dynamics of Reed Communities in Heterogeneous Habitats in Liaohe River Delta	*Phragmites australis*
70	Growth Traits of *Miscanthus sinensis* under Different Habitats in Benxi，Liaoniang Province	*Miscanthus sinensis*
71	The clonal growth of *Hedysarum mongolicum* and its water physiological effects under different habitats in the Mu Us Sandy Land	*Hedysarum mongolicum*
72	Responses of Functional Traits of *Ammopiptanthus mongolicu*s to Soil Moisture	*Ammopiptanthus mongolicus*
73	Community Structure and Species Diversity of *Atraphaxis bracteata* In Minqin Liangucheng Nature Reserve	*Atraphaxis bracteata*
74	Leaf Traits of 110 Landscape Plant	*Cinnamomum*

	Species in Nanchang	*camphora*、*Photinia* × *fraseri*、*Osmanthus fragrans*、*Lagerstroemia indic*、*Rhododendron simsii Planch*、*Nerium oleander*、*Camellia japonica*、*Hibiscus syriacus*
75	Leaf Functional Traits of *Phragmites australis* in Inland River Wetlands and Their Responses to Soil Environmental Factors	*Phragmites australis*
76	Comparisons of *Leymus chinensis* Characters in Different Habitats in the Inner Mongolian Plateau	*Leymus chinensis*
77	Clonal Architecture and Ramet Population Characteristics of *Leymus chinensis* from Different Habitats in the Xilin River Watershed	*Leymus chinensis*
78	Response of Effective Components and Growth Physiological Characteristics of *Periploca sepium* to Ecological Factors in Different Habitats in Ningxia	*Periploca sepium*
79	Clonal Reproductive Property and Community Characteristics of Invasive Species *Coreopsis lanceolata*	*Coreopsis lanceolata*
80	Functional Traits Variation Regularity of Invasive Plant *Gaura parviflora*	*Gaura parviflora*
81	Module Biomass and Allocation Characteristics of Invasive Plant *Tagetes minuta* Populations in Different Habitats	*Tagetes minuta*
82	Analysis of Growth-related Traits of Invasive *Cenchrus pauciflorus* in Heterogeneous Habitats	*Cenchrus pauciflorus*

83	Phenotypic Plasticity of *Distylium chinense* Leaves in Relation to Soil Environmental Factors in Heterogeneous Habitats in the Three Gorges Reservoir Region	*Distylium chinense*
84	Adaptation of Xylem Structure and Function of Three Gymnosperms to Different Habitats	*Pseudolarix amabilis*、*Cunninghamia lanceolata*、 *Cedrus deodara*
85	Distribution Patterns and Functional Diversity of Woody Plants in Abies Forests across Different Environmental Gradients in Sejila Mountain	*Abies georgei var. smithii*
86	Response of Leaf Functional Traits of *Calligonum mongolicum* to Habitat Changes	*Calligonum mongolicum*
87	Plasticity of Morphological and Physiological Traits in *Caragana korshinskii* under Different Habitats of the Water-Wind Erosion Crisscross Region of the Loess Plateau	*Caragana korshinskii*
88	Study of *Pinus tabulaeformis* Plantation Community Characteristics and Regeneration in Different Habitats of Huanglong Mountain Region, Shaanxi	*Pinus tabulaeformis*
89	Impacts of Varying Habitats on the External Morphological Structure of *Ageratina adenophora* Leaves	*Ageratina adenophora*
90	Phenotypic Plasticity of A Dominant Bamboo Species(Phyllostachys glauca) in Limestone Mountain in Northwest of Jiangxi Province	*Phyllostachys glauca*
91	Phenotypic Variation and Molecular Ecology of *Phragmites australis* in Heterogeneous Habitats of the Songnen Meadow	*Phragmites australis*
92	Phenotypic Plasticity of *Phragmites australis* Population Leaves in Heterogeneous Habitats of the Songnen Meadow	*Phragmites australis*

93	Adaptability of Leaf Functional Traits to Environment of *Nitraria tangutorum*	*Nitraria tangutorum*
94	Morphological and Physiological Variation and Seed Germination Physiology of *Dysosma versipellis*	*Dysosma versipellis*
95	Study on Population Distribution, Morphological Characteristics and Physiological-Biochemical Characteristics of *Dactylis glomerata* in East and West Sections of Tianshan Mountain North Slope	*Dactylis glomerata*
96	The Phenotypic Variation and Environmental Adaptability among Different Geographical Populations of *Amaranthus palmeri* in China	*Amaranthus palmeri*
97	Root Traits of Seven *Stipa* Species and Their Relations with Environmental Factors in Temperate Grasslands	*Stipa spp.* （*including S. baicalensis, S. grandis, S. krylovii, S. glareosa, S. breviflora, S. gobica, S. bungeana*）
98	Stoichiometric Characteristics and Adaptation Mechanisms of Typical Karst Forest Plants in Microhabitats	*Cyclobalanopsis phanera*、*Acer wangchii*、*Lindera communis*、*Cornus wilsoniana*、*Platycarya strobilacea*、*Zenia insignis*、*Nandina domestica*、*Brassaiopsis glomerulata*、*Miliusa sinensis*、*Murraya exotica*
99	Adaptability and Reproductive Characteristics of Invasive Plant *Mikania micrantha* under Heterogeneous Environments	*Mikania micrantha*
100	Effects of Water Levels in Heterogeneous Habitats on Sexual Reproductive Allocation of *Deyeuxia angustifolia*	*Deyeuxia angustifolia*
101	Characteristics and Assessments of Functional Traits and Anticipated Performances among Urban	*Sophora japonica*、*Aesculus chinensis*、*Koelreuteria paniculata*

	Summer-Flowering Tree Species in Heterogeneous Habitats	、*Hibiscus syriacus*、*Rosa chinensis* 、*Sambucus williamsii*
102	Population Characteristics and Growth Status of *Impatiens macrovexilla* in Heterogeneous Habitats	*Impatiens macrovexilla*
103	Response and Adaptability of Growth, Leaf Color and Photosynthetic Characteristics to Heterogeneous Habitats of Introduced *Acer palmatum*	*Acer palmatum*
104	Clonal Architecture and Ramet Population Characteristics of *Stipagrostis pennata* in Different Environments	*Stipagrostis pennata*
105	Quantitative Characteristics of the Ramet Module of *Phragmites australis* Populations in Heterogeneous Habitats in the Zhalong Wetland National Reserve	*Phragmites australis*
106	Growth Analysis of the Ramets of *Phragmites australis* in Different Habitats in Zhalong Wetland	*Phragmites australis*
107	Variation in Functional Traits of *Phragmites australis* in Different Habitats and Their Responses to Soil Factors in Zhalong Wetland	*Phragmites australis*
108	The Clonal Architecture of *Typha orientalis* under Different Water Levels in Marsh Wetlands	*Typha orientalis*
109	Interaction Between Plant Leaf Functional Traits and Atmospheric Particle Retention	*Loropetalum chinense var. rubrum Yieh*、*Rhododendron × pulchrum Sweet*、*Euonymus japonicus 'Aurea-marginatus'*、*Photinia × fraseri Dress*、*Osmanthus fragrans var. thunbergii Makino*、*Broussonetia papyrifera (L.) L'Hér. ex Vent.*、*Alternanthera philoxeroides (Mart.)*

		Griseb.、*Hydrocotyle vulgaris L.*
110	Study on the Functional Traits of Common Woody Plants in Chongqing Mountain Park	*Loropetalum chinense*、*Prunus salicina*、*Sloanea sinensis*、*Distylium racemosum*、*Juglans regia*、*Melia azedarach*、*Gleditsia sinensis*、*Firmiana simplex et al.*
111	Growth Characteristics of Landscape Trees in Chongqing and Their Response to Habitat	*Acer palmatum*、*Cinnamomum japonicum*、*Cinnamomum camphora Presl*、*Loropetalum chinense var. Rubrum*、*Pittosporum tobira*、*Malus spectabilis*、*Prunus salicina*、*Prunus cerasifera f. atropurpurea*、*Cercis chinensis*、*Magnolia denudata*、*Armeniaca mume*、*Amygdalus persica var. persica f. rubro-plena*、*Amygdalus persica var. persica f. atropurpurea*、*Amygdalus persica*
112	The Relationship between Plant Functional Traits and Soil Physicochemical Properties in the Riparian Zones of Downtown Chongqing	*Cynodon dactylon*、*Hemarthria altissima*、*Alternanthera philoxeroides*、*Humulus scandens*、*Echinochloa crusgalli*、*Commelina communis*
113	Comparison of the Growth of *Tilia amurensis* in Natural and Urban Environments	*Tilia amurensis*
114	Differences in Anatomical Structure and Hydraulic Function of Xylem in Branches of Angiosperms in Field and Garden Habitats	*Acer buergerianum*、*Cyclobalanopsis glauca*、*Ligustrum lucidum*
115	Divergent Adaptative Mechanism	*Betula Platyphylla*

	of Modules Functional Traits of *Betula Platyphylla* in Heterogeneous Habitats in Wudalianchi Volcanoes	
